# Compartment‐specific ^13^C metabolic flux analysis reveals boosted NADPH availability coinciding with increased cell‐specific productivity for IgG1 producing CHO cells after MTA treatment

**DOI:** 10.1002/elsc.202100057

**Published:** 2021-11-09

**Authors:** Andy Wiranata Wijaya, Natascha Verhagen, Attila Teleki, Ralf Takors

**Affiliations:** ^1^ Institute of Biochemical Engineering University of Stuttgart Stuttgart Germany

**Keywords:** 13C MFA, cell‐specific productivity, CHO, MTA, NADPH

## Abstract

Increasing cell‐specific productivities (CSPs) for the production of heterologous proteins in Chinese hamster ovary (CHO) cells is an omnipresent need in the biopharmaceutical industry. The novel additive 5′‐deoxy‐5′‐(methylthio)adenosine (MTA), a chemical degradation product of S‐(5′‐adenosyl)‐ʟ‐methionine (SAM) and intermediate of polyamine biosynthesis, boosts the CSP of IgG1‐producing CHO cells by 50%. Compartment‐specific ^13^C flux analysis revealed a fundamental reprogramming of the central metabolism after MTA addition accompanied by cell‐cycle arrest and increased cell volumes. Carbon fluxes into the pentose‐phosphate pathway increased 22 fold in MTA‐treated cells compared to that in non‐MTA‐treated reference cells. Most likely, cytosolic ATP inhibition of phosphofructokinase mediated the carbon detour. Mitochondrial shuttle activity of the α‐ketoglurarate/malate antiporter (OGC) reversed, reducing cytosolic malate transport. In summary, NADPH supply in MTA‐treated cells improved three fold compared to that in non‐MTA‐treated cells, which can be regarded as a major factor for explaining the boosted CSPs.

**Abbreviations**
^13^C MFA: ^13^C Metabolic Flux Analysis
CHO: Chinese Hamster Ovary
MTA: 5′‐deoxy‐5′‐(methylthio)adenosine
VCD: viable cell density

## 1 INTRODUCTION

Biopharmaceutical markets are steadily increasing worldwide; monoclonal antibody production in Chinese hamster ovary (CHO) cells continues to possess the largest share [1]. Product titers improved 100‐fold during the last few decades [2, 3]; however, cell‐specific productivities (CSPs) have increased only 38 fold. Optimization of CSP is a striking demand, facing the current need for intensifying bioprocesses and considering continuous production in perfusion processes. [4, 5, 6]

However, optimization of CSP requires a detailed understanding of intracellular regulation, which typically requires the concerted application of omics technologies [7], comprising genome analysis [8, 9], genome‐scale modeling [10], transcript analysis [11, 12], epigenetics [13, 14], metabolomics [15], and fluxomics [5, 16‐18].

Efforts have been undertaken for enhancing CSP by initiating growth arrest [19]. In addition to osmolarity and temperature shifts [20, 21], studies have focused on the addition of effectors such as sodium butyrate [22], valeric acid [23], glycine betaine [24], and catechin [25]. As a common observation, alterations in cell‐cycle phases coincided with increase in cell size. Both were anticipated to improve CSP [26, 27]. However, the underlying mechanisms that explain the phenotype remain fragmented. Recently, Verhagen et al. (2020a,b) [28, 29] demonstrated that cell‐cycle arrest could be achieved by exposing CHO cells to the effector 5′‐deoxy‐5′‐(methylthio)adenosine (MTA), which increased CSP by 50%.

MTA is a degradation product of the crucial methyl group donor S‐adenosyl‐methionine (SAM) and a by‐product of polyamine synthesis [30, 31]. MTA is an anticipated effector of the polyamine pathway, DNA synthesis, gene expression control, cell proliferation, lymphocyte activation, tumor development, invasiveness, apoptosis, and signaling pathways [31, 32, 33, 34, 35]. Furthermore, MTA is rapidly metabolized and the end products can be used to replenish adenosine‐based nucleotide pools [31]. Recently, Verhagen et al. (2020) [28, 29] revealed that MTA addition influenced cell size, cell cycle, and transcript levels, ultimately enhancing the IgG1 CSP of CHO‐DP12 cells. Additional studies have outlined the dose dependent effect of MTA addition [29]. Notably, MTA addition also leads to prolonged cell viabilities in the tests [29].

After preliminary identification of MTA as a boosting additive [28], further studies identified rising CSPs from 5.3 to 9.5 pg cell^–1^ day^–1^ after proper MTA addition, i.e. 0.15 pmol at 50 h [29]. Consequently, IgG1 titers increased from about 120 to 140 mg L^–1^. Hence, not only CSP but also titer improvements were achieved after MTA addition. Encouraged by these observations, this study was performed to identify the underlying metabolic mechanism leading to the production phenotype.

Compartment‐specific ^13^C metabolic flux analysis (^13^C MFA) was applied to assess the flux changes in the CHO‐DP12 culture with and without MTA supplementation. We will outline that the cells undergo significant metabolic reprogramming after MTA exposure. The rise of CSPs will be shown to correlate with increased pentose‐phosphate fluxes leading to the hypothesis that the latter should be fine‐tuned in hyperproducing cells.

**PRACTICAL APPLICATION**

Compartment‐specific ^13^C MFA is a tool for investigating cellular metabolism in mammalian cells distinguishing between different compartments such as cytosol and mitochondrion. In this study, we applied ^13^C MFA based on compartment‐specific metabolomics to unravel basic intracellular mechanisms coinciding with boosting cell specific productivities for IgG1 formation. The latter is the consequence of the addition of MTA (5′‐deoxy‐5′‐(methylthio)adenosine) to cell cultures. The technology of compartment‐specific ^13^C MFA could be well applied to investigate similar questions to optimize process and cell performance.

## 2 MATERIALS AND METHODS

### 2.1 Cell culture conditions and effector and isotopic tracer studies

An IgG1 antibody (anti‐IL‐8)‐producing CHO DP12 cell line (ATCC CRL‐1445TM) was adapted to suspension and grown in chemically defined TC‐42 medium (Xell AG, Germany). Media were supplemented with 4 mM ʟ‐glutamine and 200 nM methotrexate. Precultures were scaled up in shaking flasks (Corning, USA) at 37°C and 150 rpm (50 mm displacement) and under 5% CO_2_ with an initial Viable Cell Density (VCD) of 0.5 × 10^6^ cells mL^–1^ in a humidified incubator (Infors HT, Switzerland).

Bioreactor cultivations in batch mode were performed in a four‐fold DASGIP parallel bioreactor system DS1500ODSS (Eppendorf, Germany) with a starting volume of 1.2 L supplemented with 20 mM non‐labeled [U‐^12^C_6_] d‐glucose (d‐glucose) and an initial VCD of 0.4 × 10^6^ cells mL^–1^. The temperature was set to 37°C, and the pH was maintained at 7.1 with 1 M Na_2_CO_3_ and CO_2_ gassing, which was monitored using a conventional pH probe (Mettler‐Toledo, USA). The agitation speed was fixed at 150 rpm, and dissolved oxygen (DO) was controlled at 40% using an amperometric electrode (Mettler‐Toledo, USA). After 48 h, effector studies (EFF) were performed as biological duplicates (n = 2) by the addition of 250 μM MTA. Two reactors (n = 2) served as a reference (REF), and an equal volume of sterilized water was added to mimic dilution effects. After 60 h, isotopic tracer studies were performed through addition of ^13^C‐labeled d‐glucose, resulting in an extracellular ratio of 25% [U‐^12^C_6_]‐, 30% [1‐^13^C_1_]‐, and 45% [U‐^13^C_6_]‐d‐glucose in all reactors.

### 2.2 Quantification of viable cell density and extracellular metabolites

VCD and cellular viability were determined using an automated cell counting system (Cedex XS, Roche Innovatis, Germany) using trypan blue staining. Extracellular concentrations of d‐glucose and ʟ‐lactate were monitored using an amperometric biosensor system (LaboTRACE, Trace Analytics, Germany). Extracellular antibody concentrations (anti‐IL‐8 IgG1) were determined using an enzyme‐linked immunosorbent assay (ELISA), as described previously [21]. Extracellular MTA was quantified using the method described in Verhagen et al., 2020a [28].

Extracellular amino acid concentrations were measured using an Agilent 1200 HPLC system based on a bicratic reversed phase liquid chromatography (RPLC) method (Agilent Zorbax Eclipse Plus C18 column 250 × 4.6 mm, 5 μm equipped with an Agilent Zorbax Eclipse Plus C18 guard column 12.5 × 4.6 mm, 5 μm) with automated pre‐column derivatization and fluorometric detection [36]. We performed absolute quantification using a standard‐based external calibration and adapted sample dilutions (1 to 8) with γ‐aminobutyric acid (GABA) as an internal standard.

### 2.3 Cell size measurement and cell cycle analysis

Cell size was measured in conjuction with the VCD mesaurement using Cedex XS (Roche Innovatis, Germany). The spherical cellular geometry was assumed, and average cellular diameter was obtained as a readout. Cellular volume was calculated based on the spherical volume calculation using the average diameter as the input. Cell cycle analysis was conducted based on the method reported by Verhagen et al., 2020 [28].

### 2.4 Fast filtration sampling and extraction for metabolomics

Samples for subcellular metabolome analyses were collected at 48, 60, 61, 72, 84, 108, 132, and 168 h of cultivation time in technical duplicates from each reactor, according to a modified differential fast filtration protocol [18, 37]. Filtered and quenched cells (3 × 10^7^ cells per sample) were extracted directly (whole cell) or after selective permeabilization with digitonin (subcellular fractions), following addition of 5 mL ice‐cold 70% v/v methanol (MeOH) to the filters. Additionally, we used ʟ‐norvaline and 2‐dehydro‐3‐deoxy‐D‐gluconate 6‐phosphate (KDPG) as internal standards to monitor the stability of the filtration, extraction, and measurement. Filters with captured cells were incubated in sample cups at −20°C for 90 min, and the extraction solutions were subsequently separated using a vacuum pump. The filters and sample cups were rinsed with 2 mL of ice‐cold 50% v/v MeOH, and the collected extraction solutions were mixed with 500 μL of ice‐cold chloroform and vortexed for 20 s. The emulsion was centrifuged at 3200 × *g* for 11 min at 4°C. The upper aqueous phase was aliquoted into microcentrifuge tubes (4 × 1 mL), evaporated for 95 min at <20°C (RVC 2.33 IR, Christ), and stored at −70°C. During the entire process, the weights of the sample cups, filters, and micro‐centrifuge tubes were tracked to calculate the final extraction volumes.

### 2.5 Subcellular metabolomics using LC‐MS/MS and HPLC‐UV

Subcellular adenosine monophosphate (AMP), adenosine diphosphate (ADP), and adenosine triphosphate (ATP) concentrations in metabolic extracts were determined using an Agilent 1200 HPLC system based on a bicratic ion‐pair RPLC method (Hypersil BDS C18 column 15 cm × 4.6 mm, 3 μm equipped with a HypersilTM BDS C18 guard column 10 × 4 mm, 5 μm) and UV light (diode array detector) detection without derivatization [38]. We performed absolute quantification using a standard‐based external calibration and selected spikes of reference standard mixes to evaluate the influence of the sample matrices.

Subcellular metabolome studies were performed using an Agilent 1200 HPLC system coupled with an Agilent 6410B triple quadrupole mass spectrometer (MS‐QQQ) with an electrospray ion source (ESI). System control, acquisition, and analysis of data were performed using the commercial Mass Hunter B.06.00 software. Endogenous metabolites were separated under alkaline mobile phase conditions (pH 9.2) using bicratic hydrophilic interaction chromatography (HILIC), according to a previously described method [39] with modifications. GABA and α‐aminoisobutyric acid (AIBA) were previously added (50 μM) as internal standards and considered for monitoring instrumental fluctuations. Targeted ^13^C tracer analysis of isotopically labeled metabolite pools was performed in selected ion monitoring (SIM) mode using pre‐optimized precursor ion transitions (0.3 u) with adapted MS parameters and ESI conditions [40]. Subcellular metabolite pools were absolutely quantified through a three‐fold addition of defined amounts of non‐labeled reference standards (internal calibration). Applied multicomponent standard mixtures were adjusted according to the linear dynamic range of the targeted metabolites and previously estimated concentration levels [41]. Cytosolic depletion and mitochondrial integrity were evaluated using glucose 6‐phosphate (G6P), fructose 6‐phosphate (F6P), and *cis*‐aconitate (*cis*Aco) concentrations (Table S3).

^13^C mass isotopomer detection of highly reactive α‐keto acid pools (αKG, Pyr, Gxy) was performed using a previously established LC‐MS protocol [18] using the abovementioned platform. Previously derivatized (phenylhydrazine) metabolite isotopologue pools [42] using α‐ketovaleric acid (αKV) were separated under acidic conditions (pH 3) using an RPLC method and were detected in SIM mode with pre‐optimized settings. Determination of absolute pool concentrations was performed analogous to the abovementioned strategy.

### 2.6 Isotopic non‐stationary ^13^C metabolic flux analysis

For CHO cells, previous studies already outlined the advantages of using isotopically non‐stationary ^13^C MFA for intracellular flux estimation [16, 17, 18]. Fluxes were estimated through analyzing the time series of isotopically transient ^13^C labeling profiles in compartment‐specific metabolite pools using MATLAB version 2018a (The MathWorks, Inc., Natick, Massachusetts, USA).

### 2.7 Compartment‐specific metabolic model

The metabolic network model consisted of two compartments: the cytosol and the mitochondrion, each comprising a stoichiometric and a carbon atom transition model with 36 metabolites and 62 metabolic reactions (see Table S5). A cell density of 122 pg cell^–1^ was assumed according to the previous study [18]. Several sink reactions for amino acids and central carbon metabolites were considered to mimic anabolic demands for the *de novo* synthesis of carbohydrates, proteins, nucleic acids, and lipids [43].

### 2.8 Growth, nutrient uptake, and product formation rates

The cell‐specific growth rate was estimated using the weighted linear regression of VCDs with related standard deviations based on Equation (1).

(1) $$\frac{dc_{X}}{dt}=\;\mu c_{X}$$

By analogy, cell‐specific nutrient uptake and product formation rates were calculated:

(2) $$\frac{dc_{i}}{dt}=q_{i}\;c_{X}$$

Linear regressions were performed in MATLAB and Curve Fitting Toolbox Release 2018a.

### 2.9 Metabolite and isotopomer balancing

The balancing of metabolite *i* is described in Equation [3], assuming a (pseudo) steady state for the observation window:

(3) $$\frac{dc_{i}}{dt}=\;N\;\cdot \;v\;=\;0,$$

where **c**_i_ is the concentration vector containing all intracellular metabolites; N is the metabolic network stoichiometry matrix, containing the number of *i* metabolites and *j* reactions; and **v** is the vector containing flux distribution.

Bidirectional fluxes were defined for each of the reversible reactions [44, 45], as described by Equation (4).

(4) $$\overset{⇀}{v_{j}}=\beta_{j}\;v_{j}^{net}$$

$${\overset{↼}{v}}_{j}=\overset{⇀}{v_{j}}-v_{j}^{n}et,$$

where β_j_ is the reversibility factor of reaction j (β_j_ ≥ 1).

To model transient ^13^C enrichments, the isotopomers of each metabolite *i* were balanced by Equation (5).

$$\begin{array}{ccc} & & \frac{d\left(C_{i}I_{i}\right)}{dt}=\\ & & \sum_{j=1}^{N}\left[\alpha \left(\begin{array}{c} 0\\ ⊗\\ k=1 \end{array}\left(\sum_{m=1}^{n}\mathrm{IM}M_{\;k\to m}\right)I_{k}\right)r_{j}+\left(1-\alpha \right)\left(v_{ij}r_{j}I_{i}\right)\right] \end{array}$$

with

(5) $$\alpha =\left\{\begin{array}{cc} 1, & \text{if}\;v_{ij}>0\\ 0, & \text{else} \end{array}\right.,$$

where ***c*** *_i_*, ***I*** *_i_*, and ***I*** *_k_* denote vectors of intracellular concentrations of metabolites *i* and isotopomer distribution vectors, containing molar ratios that correspond to the fractional amounts of the individual isotopologues for metabolites *i* and *k*, respectively. The isotopomer mapping matrix [46] **IMM**_k→m_ describes the isotopomer transition from reactant *k* (with *n* number of reactants) to product *m*. *v_ij_* equals the stoichiometric coefficient of metabolite *i* in reaction *j*, whose molar rate is *r_j_*. The operator ⊗ denotes element‐by‐element vector multiplication.

Furthermore, isotopomer balancing was considered for extracellular metabolites that were heavily exchanged with intracellular pools [47] (ʟ‐lactate, ʟ‐alanine, ʟ‐glutamate, and ʟ‐aspartate), as shown in Equation (6):

$$\begin{array}{ccc} & & \frac{d\left(I_{i,\mathrm{ex}}\right)}{dt}\\ & & =\frac{1}{C_{i,ex}}\;\left[\bar{C_{X}}\left({\overset{⇀}{q_{i,}}}_{ex}\cdot I_{i,\mathrm{in}}-{\overset{\leftarrow }{q_{i}}}_{ex}\cdot I_{i,\mathrm{ex}}\right)-\frac{dC_{i,ex}}{dt}I_{i,\mathrm{ex}}\right] \end{array}$$

with

$${\overset{⇀}{q_{i}}}_{ex}=\beta_{i}\;\cdot q_{i,ex}^{net}$$

(6) $${\overset{\leftarrow }{q_{i}}}_{ex}={\overset{⇀}{q_{i}}}_{ex}\;-q_{i,ex}^{net}$$

### 2.10 Simulation of ^13^C labeling experiment

The total set of ordinary differential equations (ODEs), comprising 972 equations, was solved using MATLAB 2018a based on *ode15s*.

### 2.11 Parameter estimation and uncertainty

Simulated isotopologue distributions, ***x***, were obtained through minimizing the weighted least square sum, subtracting the measured isotopologue values, ***x_m_***, as indicated in Equation (7). In total, ODEs contain 43 parameters: eight intracellular fluxes and 35 reversibility constants. Parameter fitting was performed using MATLAB 2018a with *fmincon* and *GlobalSearch* and was repeated at least 100 times starting with randomized initiation settings.

(7) $$\min_{p}\Phi =\sum \frac{{\left(x-x_{m}\right)}^{2}}{\sigma_{x}^{2}},\;$$

where ***x*** denotes vectors for the isotopologue fractions. **σ** denotes the standard deviation of each measured value.

The goodness of fit of the obtained flux distribution was assessed using the chi‐square test to determine whether the model accurately reflects the in vivo data. Statistical acceptance was assigned on the 95% confidence level (α = 0.05). The complete flux distribution results is presented in Supplementary Material 2. Furthermore, the simulated mass‐isotopomer distribution (MID) is presented in Supporting Information S4.

The measured extracellular rates, ***q_m_***, and estimated intracellular rates (optimized parameter, ***p***) were used to constrain the flux distribution, ***v***, as described in Equation (8).

(8) $$v\;={\left(\begin{array}{c} S\\ M \end{array}\right)}^{-1}\;\left(\begin{array}{c} 0\\ \left[\begin{array}{cc} q_{m} & \;p \end{array}\right] \end{array}\right),$$

where **M** is the measurement matrix containing all stoichiometric coefficients of q_m_ (measured rates [pmol cell^–1^ h^–1^]) and p (estimated parameter using mass‐isotopomer data; as intracellular fluxes [pmol cell^–1^ h^–1^]).

Parameter uncertainty was assessed using a non‐linear algorithm [48]. The method assumes that the sum of squared residual is χ^2^ distributed. Thus, the uncertainty of parameter θ was determined when optimizing the said systems with one degree of freedom (χ^2^ distributed).

## 3 RESULTS

### 3.1 MTA reduced cellular growth rate while increasing cell volume

**FIGURE 1:**
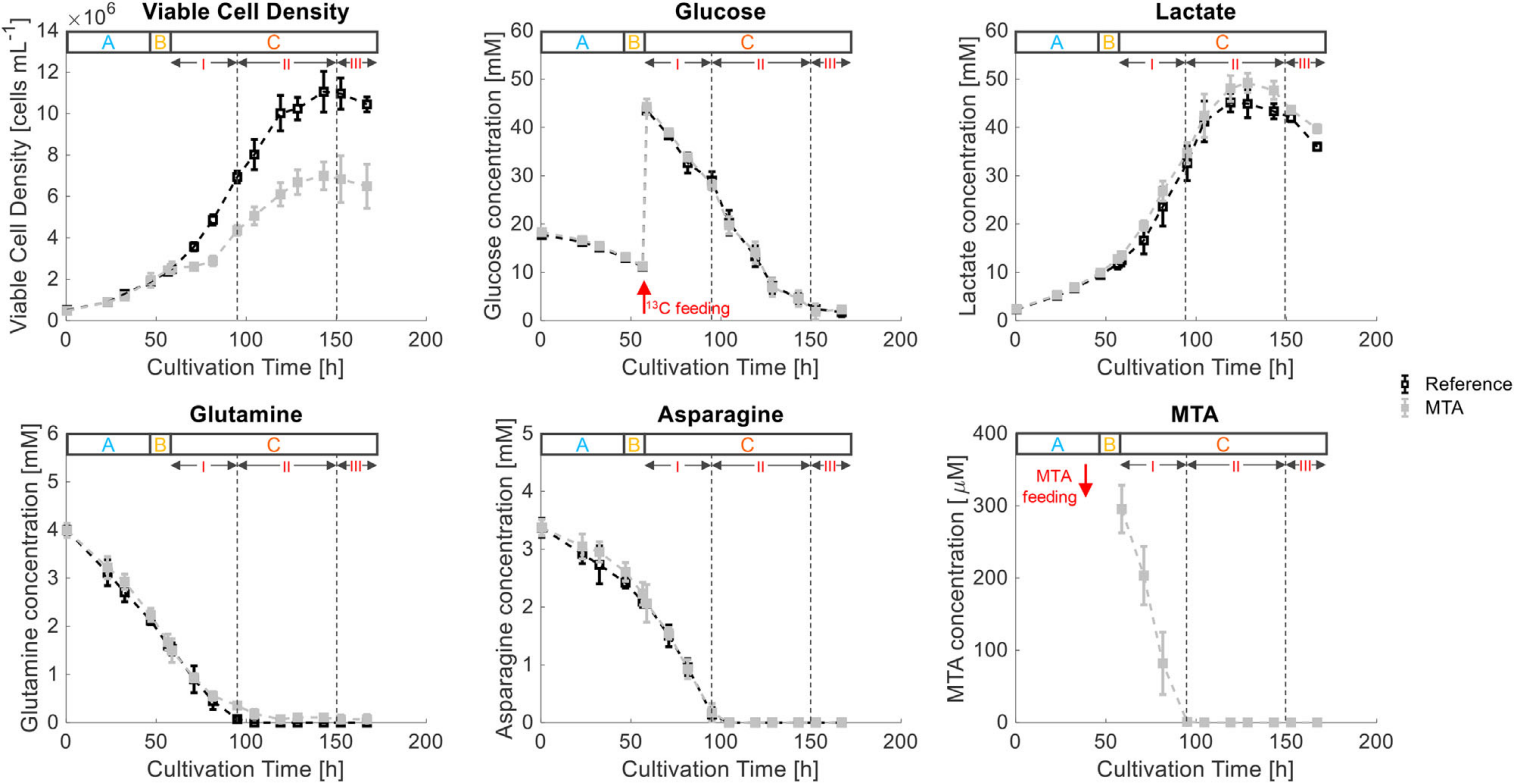
Time course of VCD [cells mL^–1^], d‐glucose [mM], ʟ‐lactate [mM], ʟ‐asparagine [mM], ʟ‐glutamine [mM], and 5′‐deoxy‐5′‐methylthioadenosine (MTA) [μM] of the reference cultures (in black) and MTA‐supplemented cultures (in grey). MTA was added after phase A (0‐48 h), at a final MTA concentration of 250 μM. Glucose labeling was initiated after phase B (48‐60 h). Phase C (60‐168 h) is divided into I: overflow; II: N‐limitation; and III: starvation. Error bars indicate the standard deviations for biological duplicates and technical replicates

**FIGURE 2:**
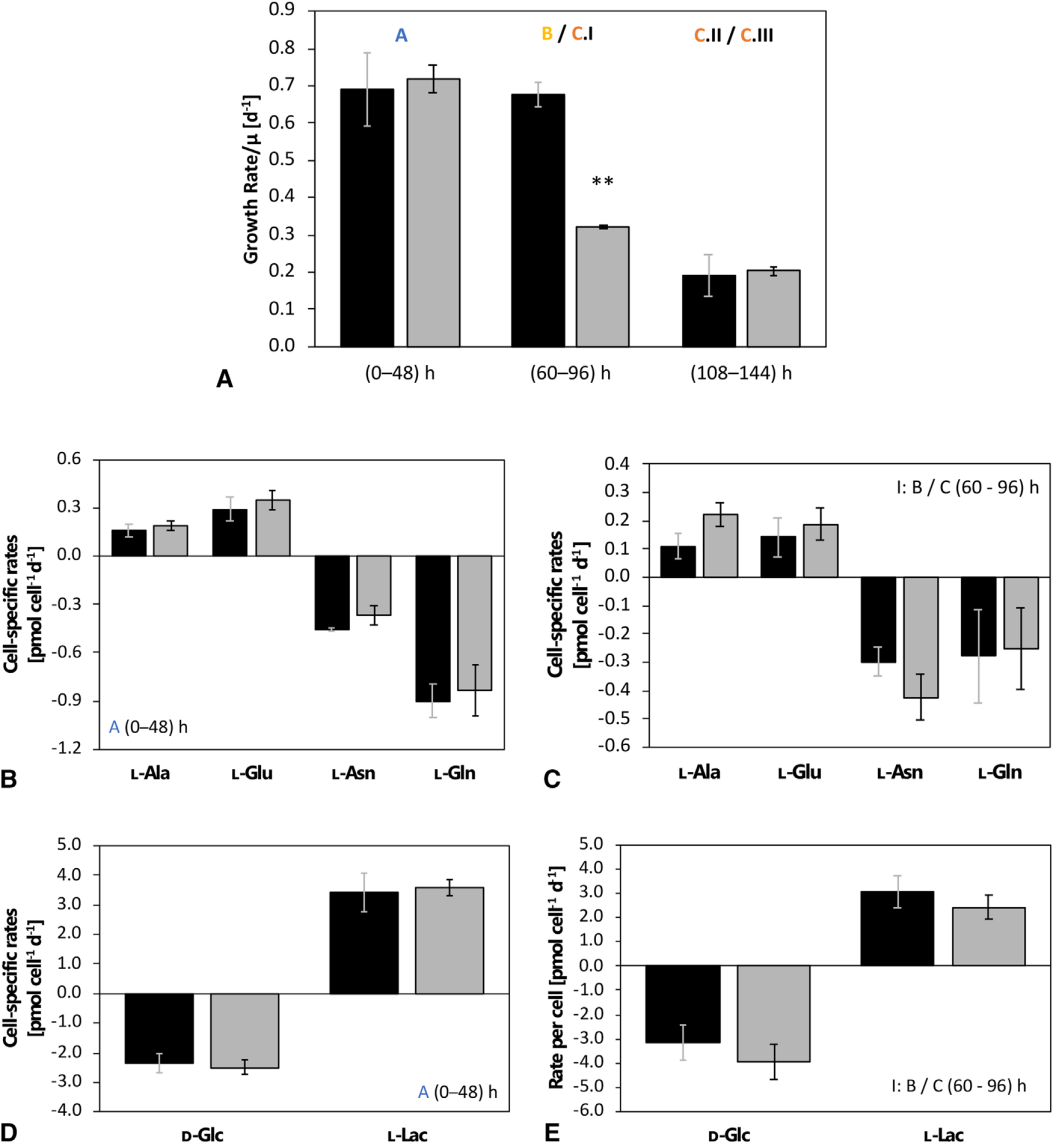
A: Growth rate per day [d^–1^] for the different cultivation phases. B: Cell‐specific uptake/secretion rates [pmol cell^–1^ d^–1^] for ʟ‐glutamine (ʟ‐Gln), ʟ‐asparagine (ʟ‐Asn), ʟ‐glutamate (ʟ‐Glu), and ʟ‐alanine (ʟ‐Ala) in overflow metabolism before MTA addition (A). C: Cell‐specific uptake/secretion rates [pmol cell^–1^ d^–1^] for ʟ‐glutamine (ʟ‐Gln), ʟ‐asparagine (ʟ‐Asn), ʟ‐glutamate (ʟ‐Glu), and ʟ‐alanine (ʟ‐Ala) in overflow metabolism after MTA addition (B/C.I). D: Cell‐specific uptake/secretion rates [pmol cell^–1^ d^–1^] for d‐glucose (d‐Glc) and ʟ‐lactate (ʟ‐Lac) in overflow metabolism before MTA addition (A). e: Cell‐specific uptake/secretion rates [pmol cell^–1^ d^–1^] for d‐glucose (d‐Glc) and ʟ‐lactate (ʟ‐Lac) in overflow metabolism after MTA addition (B/C.I). MTA‐supplemented cells (grey) compared to REF (black). MTA was added after phase A (0‐48 h) at a final MTA concentration of 250 μM. Glucose labeling was initiated after phase B (48‐60 h). Phase C (60‐168 h) is divided into I: overflow; II: N‐limitation; and III: starvation. Error bars indicate the standard deviations for biological duplicates and technical replicates. Significance was tested using one‐sided *t*‐test. **p < 0.01 *p < 0.05

During phase A (0– 48 h) cells grew under ample nutrient supply with a maximal specific growth rate of 0.705 ± 0.021 d^–1^ while consuming d‐glucose, ʟ‐asparagine, ʟ‐glutamine, and other essential amino acids (Figure 1). Metabolic overflow products, such as ʟ‐lactate, ʟ‐alanine, ʟ‐asparagine, and ʟ‐glutamate were secreted. After MTA addition during phase B, cellular growth slowed to 0.322 ± 0.003 d^–1^ and recovered after >60 h. During the first part of phase C, C.I (60‐108 h), glucose was steadily consumed. By analogy, MTA was taken up with 0.18 ± 0.037 pmol cell^–1^ d^–1^ before depletion (approximately 96 h). In accordance, the cytosolic and mitochondrial MTA levels rose, exceeding REF pool sizes by 170 and 800 times, respectively. Coinciding, the supplemented cells showed increased ʟ‐alanine and ʟ‐glutamate secretion compared to the initial phase A. d‐glucose, ʟ‐asparagine, and ʟ‐glutamine were still consumed, whereas ʟ‐lac was secreted. In contrast, reference cultures (without MTA addition) grew with an unaffected high growth rate (0.677 ± 0.031 d^–1^) (Figure 2). After 108 h, ʟ‐asparagine was depleted in all cultures. Similarly, ʟ‐glutamine depletion occurred in REF, whereas low levels were maintained in MTA‐supplemented cells. Accordingly, phase C was divided into C.I (60‐108 h) with sufficient ʟ‐asparagine and ʟ‐glutamine supply, phase C.II with ʟ‐glutamine and ʟ‐asparagine limitations (108‐144 h), and C. III as strict starvation (>144 h). For the latter, the simplifying term “nitrogen limitation” will be applied in the manuscript mirroring the important role of ʟ‐asparagine and ʟ‐glutamine as amino donors. Notably, Junghans et al. (2019) even found evidence for initiated autophagy programs under said condition. In REF, the growth was reduced to 0.258 ± 0.039 d^–1^, reflecting ʟ‐glutamine and ʟ‐asparagine limitation during C.II. Similar growth trends were observed during phase C.II for MTA‐treated cultures, with a growth rate of 0.203 ± 0.012 d^–1^ (Figure 1). Both cultures experienced strict starvation during C.III (144–168 h) with maximum VCDs of (110.5 ± 9.8) × 10^5^ cells mL^–1^ for REF and (69.9 ± 6.8) × 10^5^ for MTA‐supplemented cells (Figure 1).

**FIGURE 3:**
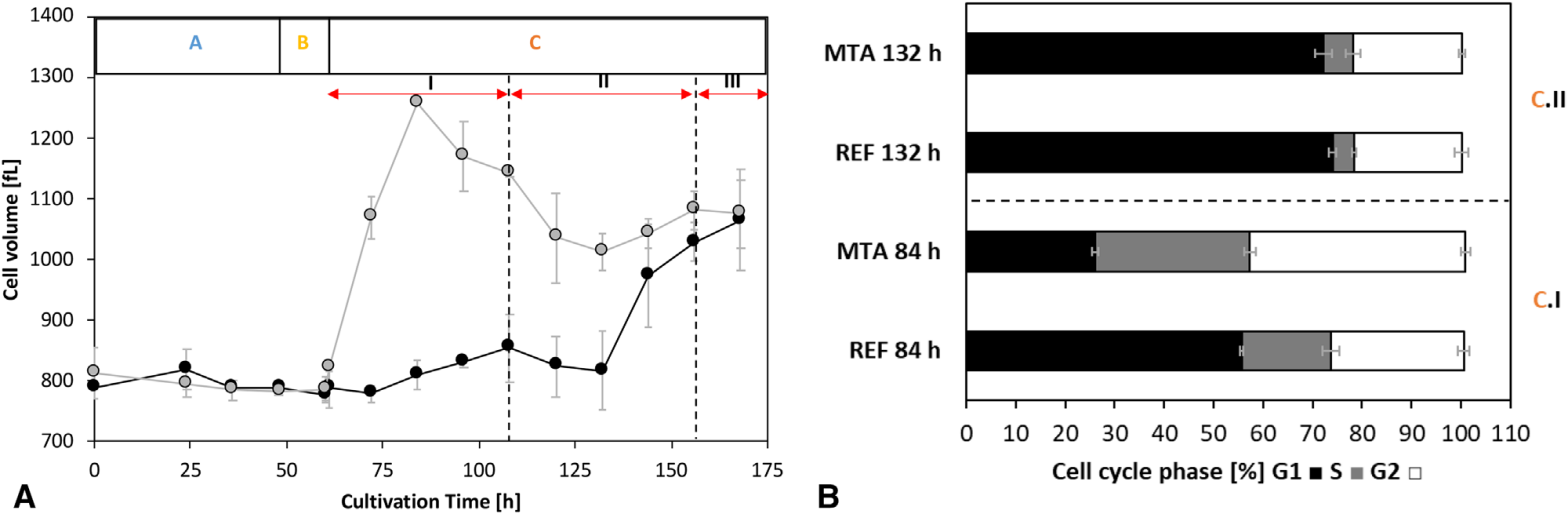
A: Time course of the cell volume [fL] of MTA‐supplemented cells (MTA: grey) and reference cells (REF: black) in the different phases. Cell volume was calculated with the assumption of a spherical cell shape. B: Cell‐cycle phase distribution [%] of MTA‐supplemented cells (MTA) and reference cells (REF) at 84 h (I: Overflow) and 132 h (II: N‐limitation). MTA was added after phase A (0‐48 h) at a final MTA concentration of 250 μM. Glucose labeling was initiated after phase B (48‐60 h). Phase C (60‐168 h) is divided into I: overflow; II: N‐limitation; and III: starvation. Error bars indicate standard deviations for biological duplicates and technical replicates

In addition to the diminished growth rate, MTA addition led to temporary increases in cell volumes. On the basis of the measured cell diameters, the volume of supplemented cells increased by 55% (84 h) and shrank by 31% during phase C.II. In contrast, the cell size of the reference cultures was stable until the end of the cultivation phase C.II. Upon initiating nitrogen starvation (phase C.III), the cell volumes rose, and the differences leveled out (Figure 3A).

The increase in cellular volumes coincided with a change in the cell‐cycle distribution after MTA addition (Figure 3B). At 84 h, the supplemented cells showed a lower G1‐fraction (MTA: 26.02 ± 0.70%; REF: 55.70 ± 0.26%) and higher S‐fraction (MTA: 31.32 ± 1.23%; REF: 17.98 ± 1.71%) and G2‐fraction (MTA: 43.60 ± 0.95%; REF: 26.88 ± 1.26%). Cells subjected to nitrogen limitation in phase C.II (132 h) exhibited equalization of the cell‐cycle phase distribution compared to the reference cultures.

### 3.2 MTA addition enhanced cell‐specific productivity and ATP availability

**FIGURE 4:**
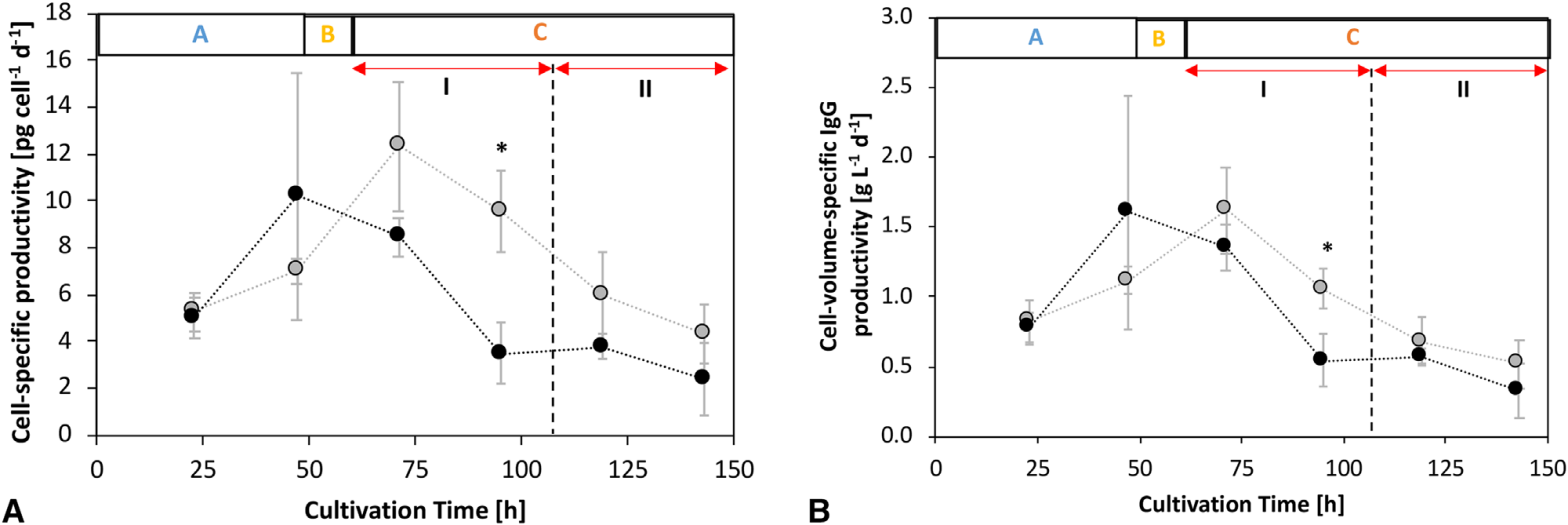
Cell‐specific IgG productivity (CSP) [pg cell^–1^ d^–1^] and cell‐volume‐specific IgG productivity (CVP) [g L^–1^ d^–1^] regarding the different cultivation phases. A: Differential CSPs over cultivation time. B: Differential CVPs over cultivation time. MTA‐supplemented cells (grey) compared to REF cells (black). MTA was added after phase A (0‐48 h) at a final MTA concentration of 250 μM. Glucose labeling was initiated after phase B (48‐60 h). Phase C (60‐168 h) is divided into I: overflow; II: N‐limitation; and III: starvation. Error bars indicate standard deviations for biological duplicates and technical replicates. Significance was tested using one‐sided *t*‐test. *p < 0.05

Figure 4A depicts cell‐specific (CSP) and cell‐volume‐specific (CVP) IgG productivities during the exponential growth phase A with abundant nutrient supply. The CSP exhibited no significant difference before MTA supplementation (e.g., differential CSP at 24 h): REF, 5.01 ± 0.88 pg cell^–1^ d^–1^; MTA, 5.26 ± 0.83 pg cell^–1^ d^–1^. Similarly, the CVP was not significantly different before supplementation (Figure 4B). Interestingly, the CSP following MTA supplementation was 170% higher (9.59 ± 1.72 pg cell^–1^ d^–1^) than that in REF (3.51 ± 1.27 pg cell^–1^ d^–1^) during C.I after transient phase B. The subsequent phases C.II and C.III show reduced CSPs for all cultures, which indicates the dominating impact of limited nutrient supply in accordance with previous study [18]. The CVPs only increased by roughly 95% (MTA: 1.05 ± 0.14 g L^–1^ d^–1^; REF: 0.54 ± 0.19 g L^–1^ d^–1^), whereas the CSPs rose by 170%. Thus, CSP improvements mirror the combinatorial effects of elevated cell volumes and metabolic changes. The latter will be investigated through ^13^C metabolic flux analysis.

**FIGURE 5:**
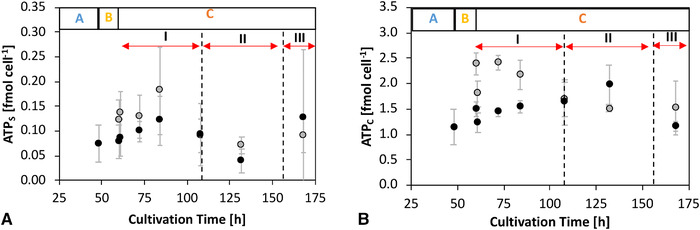
A: Time course of the ATP concentrations in the subcellular (mitochondrial, index S) compartment of MTA‐supplemented cells (MTA: grey) and reference (REF: black). B: Time course of the ATP concentrations in the cytosolic compartment (index C) of MTA‐supplemented cells (MTA: grey) and reference cells (REF: black). MTA was added after phase A (0‐48 h) at a final MTA concentration of 250 μM. Glucose labeling was initiated after phase B (48‐60 h). Phase C (60‐168 h) is divided into I: overflow; II: N‐limitation; and III: starvation. Error bars represent the standard deviations for biological duplicates and technical replicates

The cellular energy status was studied on the basis of the ATP concentrations in the cytosolic and mitochondrial compartments (Figure 5).

Considering the error bars in Figure 5A, MTA addition did not change the ATP levels in the mitochondria but significantly increased the ATP content in the cytosol. The latter peaked 12 h after MTA addition before approaching the corresponding levels in REF at the end of C.I (Figure 5B). Notably, the increase of cytosolic ATP precedes the increase in CSP (Figure 4A).

### 3.3 MTA‐treated cells detour carbon into the PPP

Isotopically transient ^13^C MFA was performed to elucidate the impact of MTA addition on central metabolism under ample nutrient supply coinciding with the highest CSPs observed in phase C.I.

Figure 6A depicts the cytosolic and mitochondrial carbon flux distributions of MTA‐supplemented and reference cultures during the exponential growth in phase C.I.

FIGURE 6A. Metabolic flux distribution of the reference and MTA‐supplemented CHO‐DP12 cells. Arrows indicate flux direction, and the thickness of the arrows indicate strength in pmol cell^–1^ day^–1^. B. Comparison of key fluxes using abbreviations as follows: phosphoglucose‐isomerase (pgi), G6P dehydrogenase (G6Pdh), endogenous glycogen exchange (fGlyco), PEP carboxykinase (PEPCk), alanine amino transferase (alt), malic enzyme (me), pyruvate carboxylase (pc), and aspartate amino transferase (ast); and C. Comparison of mitochondrial carrier activities: pyruvate/H^+^ symporter (MPC), aspartate/glutamate antiporter (AGC), citrate/malate antiporter (CIC), dicarboxylic acid carrier (DIC), glumate carrier (GC), aKG/malate antiporter (OGC), putative alanine carrier (mAla), and putative asparagine carrier (mAsn). *indicates *p* < 0.05

**Figure:**
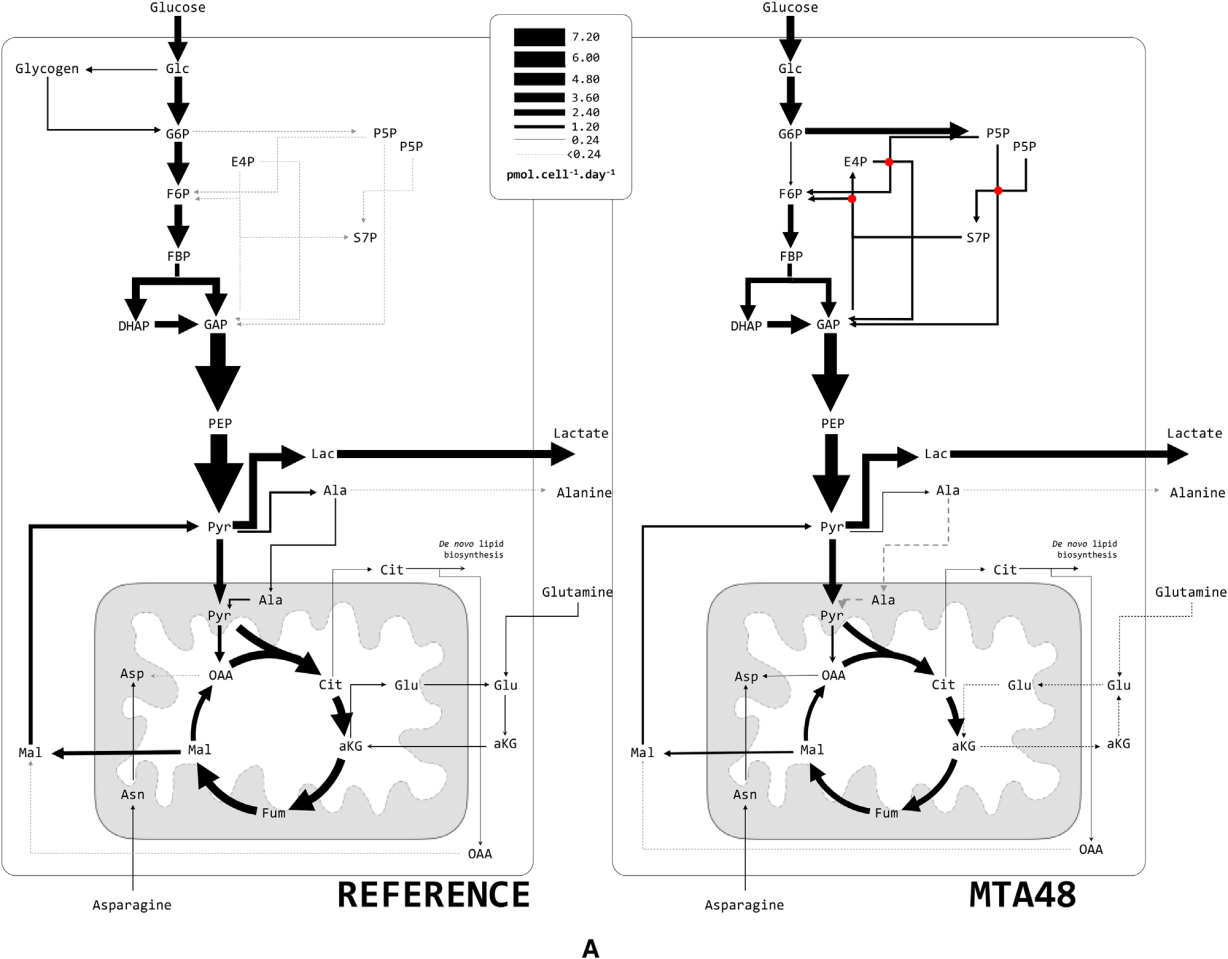

**Figure:**
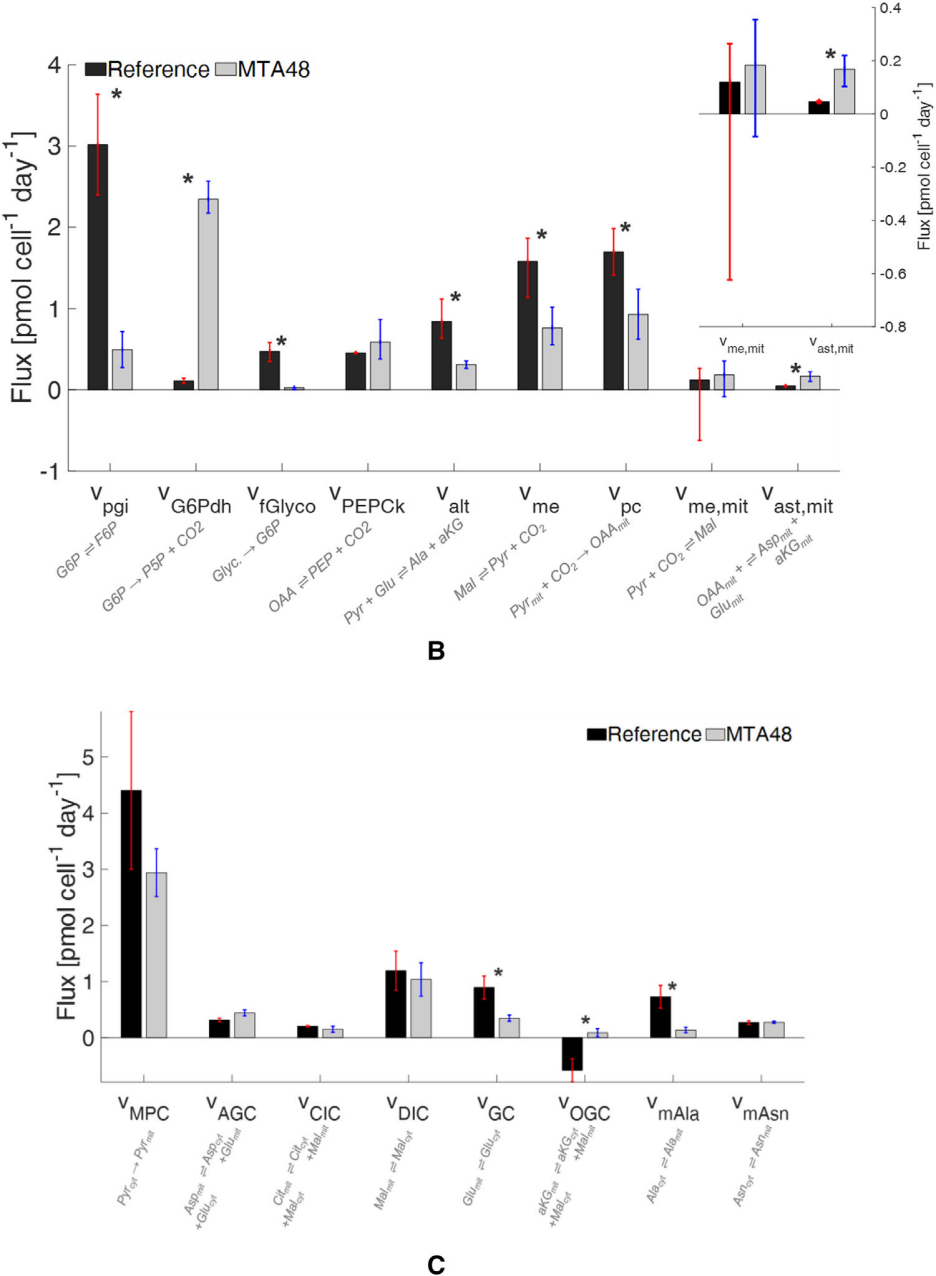

Focusing on the upper glycolysis, the CHO‐DP12 reference cultures (Figure 6A) showed a metabolic phenotype comparable to that reported by Junghans et al. (2019) [18]. Under abundant nutrient supply, approximately 18% of total d‐glucose was continuously exchanged with non‐labeled endogenous carbon storage (*v_fGlyco_*: 0.474 ± 0.109 pmol cell^–1^ d^–1^). At the same time, a remarkably low fraction of consumed d‐glucose (about 4%) was channeled into the oxidative PPP (*v_G6Pdh_*: 0.110 ± 0.032 pmol cell^–1^ d^–1^), which corresponds to the earlier findings [16, 17].

In contrast, MTA‐supplemented cells exhibited a considerably different flux distribution (Figure 6B). The labeling kinetics of G6P indicates significantly reduced carbon exchange with endogenous carbon storage compounds such as glycogen (*v_fGlyco_*: 0.02 ± 0.024 pmol cell^–1^ d^–1^). Instead, approximately 83% of consumed glucose was diverted into the oxidative PPP (*v_G6Pdh_*: 2.35 ± 0.17 pmol cell^–1^ d^–1^). This corresponds to a remarkable rise of 21‐fold in the G6Pdh flux in the MTA‐supplemented cells compared to that in the G6Pdh flux in the reference culture.

The PPP influx of MTA‐supplemented culture exceeded the anabolic ribose 5‐phosphate (R5P) requirements, i.e., precursor needs for nucleotide biosynthesis, by a factor of 166. Consequently, approximately 81% of the carbon re‐entered glycolysis via fructose 6‐phosphate (F6P) and glyceraldehyde 3‐phosphate (GAP). Interestingly, lower glycolysis (represented by GAP dehydrogenase) showed statistically similar fluxes in MTA‐supplemented (*v_GAPdh_* : 4.87 ± 0.29 pmol cell^–1^ d^–1^) and reference culture cells (*v_GAPdh_* : 6.18 ± 1.24 pmol cell^–1^ d^–1^).

### 3.4 MTA supplementation reduced net malate export from mitochondria

The compartment‐specific ^13^C MFA revealed the in vivo activity of the mitochondrial pyruvate carrier, MPC, and the other solute carriers belonging to the family 2A (SLC25A; see Figure S1). In the REF culture, the highest mitochondrial carrier activity was observed for the MPC (*v_MPC_*: 4.4 ± 1.4 pmol cell^–1^ d^–1^), supporting the earlier study [18]. The dicarboxylic acid carrier (DIC) and the glutamate carrier (GC) showed the second strongest rates of 0.89 to 1.19 pmol cell^–1^ d^–1^.

Shuttling activities for malate described the cellular status [18] well. Previous analysis of malate shuttling revealed that DIC is the key malate carrier in CHO cells. With approximately 1 pmol cell^–1^ d^–1^, malate export from the mitochondria via DIC was approximately six‐fold higher than malate export via CIC. OGC further supports the export of mitochondrial malate by importing cytosolic αKG. In total, net malate export occurs from the mitochondria to the cytosol at 1.57 ± 0.41 pmol cell^–1^ d^–1^, which implies malate‐mediated NADPH production in the cytosol [18].

MTA supplementation fundamentally altered the shuttling activities of GC, OGC, and putative alanine carrier (mAla). Remarkably, OGC transport was reverted, resulting in the export of αKG to the cytosol and import of malate into the mitochondria. Accordingly, the net malate export to the cytosol was reduced to 0.80 ± 0.31 pmol cell^–1^ d^–1^ due to the MTA supplementation. Additionally, cells exposed to MTA exhibited a reduction in *v_GC_* and *v_mAla_* to 0.35 ± 0.06 and 0.14 ± 0.05 pmol cell^–1^ d^–1^, respectively.

### 3.5 Reprogramming of NADPH production strategies while maintaining a similar NADH supply

NADPH is a vital cofactor and crucial redox partner in various cellular reactions, typically anabolic reactions [49]. In CHO‐DP12, NADPH is produced via oxidative PPP and the cytosolic malic enzyme (*me_cyt_*). The latter requires sufficient malate shuttling activity from the mitochondria via the concerted activities of MPC, CIC, and DIC in the citrate–pyruvate shuttle systems. This study identified a relatively high cytosolic malic enzyme flux (*v_me_cyt_*: 1.58 ± 0.28 pmol cell^–1^ d^–1^) in the reference culture, which is in accordance with Junghans et al. (2019) [18]. Notably, fluxes via *me_cyt_* were entirely fueled by malate exported from the mitochondria.

**FIGURE 7:**
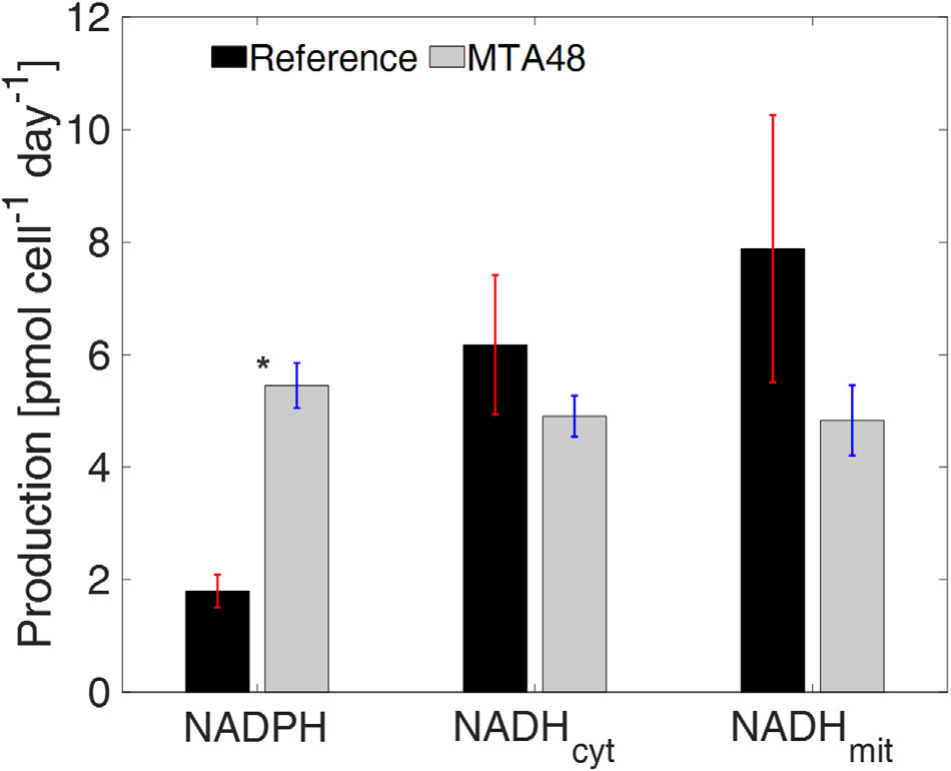
Comparison of NADPH, NADH_cyt_, and NADH_mit_ production in reference culture and MTA‐treated culture. *Indicates a significant difference (*p* < 0.05)

In the MTA‐supplemented cultures, *me_cyt_* activity (0.76 ± 0.21 pmol cell^–1^ d^–1^) was reduced by 43%, mirroring the reduction of shuttling activities. Consequently, the NADPH supply fundamentally differed between the REF and MTA‐treated cells (Figure 7). In the reference culture, NADPH supply via cytosolic malic enzyme (*me_cyt_*) comprises 88% of the total production compared to 14% in the culture treated with MTA. Despite severe reduction *in me_cyt_* activity, the MTA‐treated culture produced approximately three‐fold more NADPH than REF through amplification of the NADPH formation via PPP. Notably, PPP‐mediated NADPH formation was approximately 4.69 ± 0.34 pmol cell^–1^ d^–1^, reflecting a 21‐fold increase compared to REF. Regarding NADH supply, no statistically significant differences were observed between the REF and MTA‐treated cells (Figure 7).

### 3.6 Glutamine and alanine metabolism

ʟ‐glutamine uptake rate was reduced in the MTA‐treated cells (0.16 ± 0.03 pmol cell^–1^ day^–1^) compared to the reference culture (0.22 ± 0.01 pmol cell^–1^ day^–1^). Nevertheless, ʟ‐glutamine catabolic rates remained similar in both cultures (REF: 0.17 ± 0.01 pmol cell^–1^ day^–1^; MTA treated: 0.14 ± 0.03 pmol cell^–1^ day^–1^). In REF, ʟ‐glutamine was taken up and initially catabolized in the cytosol via glutaminase (Figure 6A). Next, the intermediate product (ʟ‐glutamate) was catabolized via cytosolic amino‐transferases yielding αKG, which fueled into the CAC via the Mal/αKG symporter (OGC). This observation is consistent with the observations of Junghans et al. (2019) [18]. Interestingly, cultures treated with MTA showed a variation: cytosolic glutamate was imported directly into the mitochondria via AGC, and it did not require further cytosolic deamination to αKG.

Cellular secretion of ʟ‐alanine was significantly higher in the MTA‐supplemented cells than in the REF (Figure 2). In contrast, intracellular ʟ‐alanine formation mirrors cytosolic alanine‐aminotransferase (*alt_cyt_*) activities *v*_alt_cyt_, which were lower in the MTA‐treated cells (0.31 ± 0.05 pmol cell^–1^ day^–1^) than in REF (0.84 ± 0.20 pmol cell^–1^ day^–1^).

## 4 DISCUSSION

The addition of MTA and its boosting effect on the CSP in CHO‐DP12 cells was first studied by Verhagen et al. (2020) [28, 29]. MTA plays an essential role in mammalian cell metabolism, especially in polyamine synthesis [30, 31]. In this pathway, MTA serves as an intermediate decarboxylation product of SAM to produce spermidine and spermine. High MTA levels are anticipated to inhibit polyamine biosynthesis, finally inducing cell‐cycle arrest [50]. Besides, MTA is known to impact gene expression patterns, including apoptosis and cell proliferation in various mammalian cells [32, 35]. In summary, MTA serves as a multi‐level regulatory compound that exerts genetic, epigenetic, and metabolic control.

The reduction in spermidine and spermine synthesis after MTA addition is known to induce S‐phase arrest in CHO cells [51]. This study confirmed the cytostatic effect, as displayed in Figure 1. MTA addition significantly reduced the maximum VCD and cell‐specific growth rate (Figure 2A). Concomitantly, cellular volume increased (Figure 3A), cell‐cycle distribution changed (Figure 3B), and CSP (q_mAb_) (Figure 4) increased following MTA addition. Cellular fractions in the S‐ and G2‐phases were temporarily increased 36 h after MTA addition, while the number of cells in G1‐phase decreased (Figure 3B). The increase in q_mAb_ (Figure 4) 48 h after MTA addition might be attributed to cell‐cycle arrest, as previously reported by independent studies [20, 21]. Notably, CSP raise is not limited to a distinct cell‐cycle phase.

Moreover, increase in cell volumes [52, 53] have been reported to enhance CSP. Although rising cell volumes were observed in our study (Figure 3A), comparably low CVPs suggested that post‐MTA CSP improvements were not caused by rising cell volumes alone (Figure 4). Indeed, the CVPs of MTA‐treated cells remained significantly higher compared to the REF observation (Figure 4). However, fundamental reorganization of metabolism in arrested cells is supposed to be the key factor explaining rising CSPs [54].

Metabolic rearrangements were studied via ^13^C labeling analysis. Thus, the approach complements earlier studies by Verhagen et al. (2020) [28, 29], which focused on the SAM/MTA interplay and transcriptional responses after MTA feeding. Particularly, compartment‐specific flux analysis was applied according to a previously reported protocol [18] for determining the putative impact of NADPH supply. Moreover, compartment‐specific metabolomics essentially enabled flux tracking inside cellular compartments to unravel the putative impact of trans‐compartment shuttling activities. Both data are qualified as important information per se.

MTA addition apparently influences the energetic status of the cells, which is reflected by an increase in cytosolic ATP pools (Figure 5). We anticipate that anabolic ATP needs are reduced in MTA treated cells as growth rates slowed down. Consequently, ATP pools increased meanwhile. Interestingly, similar observations of ATP rise were made in growth/cell‐cycle arrested CHO cells [11]. The temporal rise in ATP levels likely inhibits phosphofructokinase (PFK) activity, considering the equally high ATP inhibition constants [55, 56, 57]. This metabolic inhibition may explain why fluxes through PFK are lower following MTA addition compared to REF cultures (Figure 6).

Moreover, Moreadith and Lehninger (1984) showed that malic enzyme activity is inhibited by high ATP levels [58]. Consequently, enhanced flux into the oxidative PPP is likely to reflect ATP‐mediated metabolic inhibition of PFK and malic enzyme. The latter caused a reduction in NADPH formation, which was counterbalanced by an increase in PPP fluxes, ultimately achieving elevated NADPH production (Figure 7). The coincidence of metabolic rearrangement and cell‐cycle arrest following MTA addition is consistent with the findings of Vizán et al. (2009) [59]. The authors additionally observed a relatively high oxidative PPP activity during the late G1 and S phases, suggesting that more precursors for nucleotide biosynthesis are needed during the S‐phase in the cell cycle.

In addition, compartment‐specific flux analysis revealed that OGC flux was reversed in MTA‐supplemented cultures compared to REF. Whereas OGC exported malate into the cytosol in REF, OGC imported cytosolic malate in MTA‐treated cultures. Nevertheless, malate net export into the cytosol continued to be realized via DIC. *v_DIC_* was comparable with that of the reference; however, lack of OGC contribution reduced the net malate export into the cytosol following MTA addition. Consequently, cytosolic malate was adapted to the decrease in cytosolic malic enzyme activity. Notably, the reversion of OGC reverted the flux of the counter ion αKG. Specifically, instead of importing αKG in the REF culture, αKG was exported into the cytosol in the MTA‐treated cultures.

In REF, ʟ‐glutamine was deaminated via cytosolic glutaminase and glutamate dehydrogenase before importing αKG into the mitochondria. Because OGC shuttling is reversed, the MTA‐treated cells require an alternative pathway to fuel the ʟ‐glutamine intermediate into CAC. Nevertheless, ʟ‐glutamine was deaminated via cytosolic glutaminase. Next, ʟ‐glutamate was imported into the mitochondrion via the amplified activities of the shuttle AGC. Subsequent deamination to αKG occurred in the mitochondrion. Interestingly, the potential export of mitochondrial ʟ‐glutamate via GC slowed down to ensure sufficient ʟ‐glutamate supply inside the mitochondrion.

The compartment‐specific ^13^C MFA additionally revealed that alanine amino‐transferase (*alt_mit_* and *alt_cyt_*) activities were reduced in the MTA‐treated culture. As ʟ‐glutamate was net imported into the mitochondria, the formation of ʟ‐alanine as the amino receptor from ʟ‐glutamate in the cytosol was significantly reduced (Figure 6). Consequently, less ʟ‐alanine was imported into the mitochondria (reduced *m_ala_*). Furthermore, the substrate αKG of *alt*_mit_ was shuttled out of the mitochondria in exchange with malate and *v_alt,_*_mit_ was finally reduced. In essence, the activity of intracellular ʟ‐alanine metabolism was lowered, which led to increased ʟ‐alanine secretion into the medium.

Both scenarios reflect the changes in ʟ‐glutamine metabolism; they are essentially consequences of the rearrangement of glycolytic fluxes following MTA addition. Specifically, the temporal ATP rise was likely to inhibit PFK and cytosolic malic enzyme activities, which resulted in strongly amplified PPP fluxes for improving NADPH formation. Cytosolic malic enzyme lost its dominant role as a key NADPH supplier, which resulted in reduced mitochondrial malate exports and ʟ‐glutamine metabolic rearrangements, as described above.

Junghans et al. (2019) previously outlined the high sensitivity and positive correlation between NADPH supply and CSP for monoclonal antibody formation in CHO cells [18]. Their compartment‐specific metabolome analysis revealed cytosolic malic enzyme as the key NADPH provider in growing cells. Previously, the crucial role of malic enzyme as the major NADPH source was anticipated by Ahn and Antoniewicz (2011) and Templeton et al. (2013) [16, 17]. Sengupta et al. (2011) analyzed fluxes in late‐stage non‐growing CHO and disclosed increasing fluxes into the PPP, apparently for NADPH formation [60]. Furthermore, Tuttle et al. (2000) observed a 200‐fold increase in G6PDH activity under oxidative stress conditions [61].

The key reason for the occurrence of fundamental metabolic reprogramming in arrested cells remains open and deserves future studies. However, a beneficial link to heterologous protein formation exists. Flux balance analysis using the simplified metabolic model [62] published by Verhagen et al. (2020c) considered that the maximum NADPH supply should be supported by high cytosolic malic enzyme flux and high oxidative PPP activity (Supplementary Material 3). Interestingly, MTA treatment provides an optimal flux distribution to maximize the NADPH supply. This observation opens the door for bioprocess optimization, including investigation of the impact of serial MTA bolus shots to extend the phase of high‐level CSPs.

Alternately, metabolic engineering strategies may focus on redirecting fluxes for the highest NADPH supply. Balsa et al. (2020) evaluated deletions of *G6PDH*, *ME1*, and *IDH1* to foster PPP fluxes and NADPH formation [63]. However, *G6PDH* deletion resulted in higher oxidative stress, slowed down growth, and even led to cell death. This finding is in accordance with the observations of Tuttle et al. (2000) [61]. Till date, deletions of *ME1* or *IDH1* have not been reported to induce anticipated NADPH improvements.

## 5 CONCLUSIONS

The current study merges recent approaches of compartment‐specific metabolomics and non‐stationary ^13^C flux analysis to investigate the impact of MTA addition on monoclonal antibody‐producing CHO cells. Complementing the studies of Verhagen et al. (2020), improvement in CSPs was determined to be tightly linked with cellular arrest and reprogramming of metabolism. MTA addition initiates a cascade of regulatory responses comprising cell proliferation, transcription, and metabolic control, still keeping cell viabilities on a high level. Most likely, the first is the consequence of MTA‐mediated feedback inhibition in polyamine biosynthesis. The resulting accumulation of ATP impairs glycolytic and malic enzyme fluxes, finally enabling significantly enhanced NADPH supply via boosting PPP activity. This knowledge should be exploited via future bioprocess development and metabolic engineering studies for improving the CSP for next‐generation CHO hosts. Besides, it may illustrate the close network of cellular responses ranging from cell proliferation to reverting mitochondrial shuttle activities because of MTA addition to a CHO culture.

**Nomenclature**
C_x_
: Viable cell density (VCD) (cell/mL)
C_i_
: Extracellular concentration of metabolite *i* (mmol/L)
q*_i_*
: Extracellular production/consumption rate of *i* (pmol/cell/day)
*S*
: Stoichiometric matrix of the metabolic model ([‐])
*v_i_*
: Intracellular flux of reaction *j* (pmol/cell/day)
I: metabolite pool size (fmol/cell)
*x*
: simulated mass isotopomer species ([‐])
*x_m_*
: measured mass isotopomer species ([‐])

**Greek symbols**
μ: cell‐specific growth rate (h^–1^)
β_j_
: flux reversibility constant of reaction *j* ([‐])
σ: standard deviation ([‐])

## CONFLICT OF INTEREST

The authors have declared no conflict of interest.

## Supporting information

Supporting information.

Supporting information.

Supporting information.

Supporting information.

## Data Availability

The datasets supporting the conclusions of this article are included within the article and its additional files.
