# Supplementary material for: Compartment‐specific 13C metabolic flux analysis reveals boosted NADPH availability coinciding with increased cell‐specific productivity for IgG1 producing CHO cells after MTA treatment
Source: Eng Life Sci. 2021 Nov 9;21(12):832–47. doi: 10.1002/elsc.202100057 (PMC8638276; doi:10.1002/elsc.202100057)
Supplement: Supplementary file 1 — Supporting information. [file ELSC-21-832-s003.docx]

**Supplemental Material 1**

Table S1. List of chemicals

| **Chemical** | **Supplier** |
| --- | --- |
| L-glutamine | Carl Roth GmbH & Co. KG, Germany |
| methotrexate | Sigma-Aldrich, Germany |
| D-glucose [U-^12^C] | Carl Roth GmbH & Co. KG, Germany |
| D-glucose [U-^13^C] | Cambridge Isotope Laboratories, Inc, US |
| D-glucose [1-^13^C_1_] | Sigma-Aldrich, Germany |

Table S2. Full names of abbreviated metabolites.

| 1 | 1,3-BPG | 1,3-bisphosphoglycerate | 27 | Gly | glycine |
| --- | --- | --- | --- | --- | --- |
| 2 | 2/3-PG | 2/3-phosphoglycerate | 28 | Glyco | glycogen |
| 3 | AcCoA | acetyl coenzyme A | 29 | Gxy | glyoxylate |
| 4 | ADP | adenosine diphosphate | 30 | IsoCit | isocitrate |
| 5 | aKG | α-ketoglutarate | 31 | Isoleu | isoleucine |
| 6 | aKV | α-ketovalerate | 32 | KDPG | 2-keto-3-deoxy-6-phosphogluconate |
| 7 | Ala | alanine | 33 | Lac | lactate |
| 8 | AMP | adenosine monophosphate | 34 | Leu | leucine |
| 9 | Arg | arginine | 35 | Mal | malate |
| 10 | Asn | asparagine | 36 | Met | methionine |
| 11 | Asp | aspartate | 37 | MMA | mono-methyl-adipate |
| 12 | ATP | adenosine triphosphate | 38 | NAD | ox nicotinamide adenine dinucleotide |
| 13 | cisAco | cis-aconitate | 39 | NADH | red nicotinamide adenine dinucleotide |
| 14 | Cit | citrate | 40 | NADP | ox nicotinamide adenine dinucleotide phosphate |
| 15 | CoA | Coenzyme A | 41 | NADPH | red nicotinamide adenine dinucleotide phosphate |
| 16 | DHAP | dihydroxyacetone phosphate | 42 | Nva | norvaline |
| 17 | F16bp | fructose 1,6-bisphosphate | 43 | OAA | oxaloacetate |
| 18 | F6P | fructose 6-phosphate | 44 | PEP | phosphoenolpyruvate |
| 19 | FAD | flavin adenine dinucleotide | 45 | Pro | proline |
| 20 | FADH_2_ | FAD (hydroquinone form) | 46 | Pyr | pyruvate |
| 21 | Fum | fumarate | 47 | R(u)5p | rib(ul)ose 5-phosphate |
| 22 | G6P | glucose 6-phosphate | 48 | Ser | serine |
| 23 | GAP | glyceraldehyde 3-phosphate | 49 | Suc | succinate |
| 24 | Glc | glucose | 50 | Thr | threonine |
| 25 | Gln | glutamine | 51 | Tyr | tyrosine |
| 26 | Glu | glutamate | 52 | Val | valine |

**Sub-cellular metabolomics correction factor**

Correction factor for the validation of the differential fast-filtration method for whole-cell and subcellular metabolome analyses. The compartment-specific internal standards G6P, F6P (cytosol) and cisAco (mitochondria) were used to evaluate mitochondrial integrity and cytosol depletion. The correction factor was separately calculated for REF and MTA supplemented cells as in Junghans et al.(2019). The measured whole-cell (w-c) and subcellular(s) concentrations of the compartment-specific internal standards G6P, F6P (cytosol) and cisAco (mitochondria) were compared to each other with the following formula to demonstrate the separation by the fast-filtration method:

$$\frac{{cisAco}_{w-c}}{{cisAco}_{s}}*(1-0.5*\frac{{G6P}_{s}}{{G6P}_{w-c}}-0.5*\frac{{F6P}_{s}}{{F6P}_{w-c}})$$

Standard deviation of all intracellular samples. Significance was tested with a one-sided t-test.

Table S3. Correction factor for the validation of the differential fast-filtration method for whole-cell and subcellular metabolome analyses.

Table S4. Cell specific productivity (CSP) [pg cell^-1^ d^-1^] regarding the different cultivation phases: MTA supplemented cells compared to REF. MTA was added after phase A (0–48 h) installing at a final MTA concentration of 250 µM. Glucose labelling started after phase B (48–60 h). Phase C (60–168 h) is divided in I: Overflow. II: N-Limitation. III: starvation. Error bars show standard deviations of biological duplicates and technical replicates. Significance was tested with one-sided t-test. * p < 0.05.

| [pg cell^-1^ d^-1^] | A (0 - 48) h | | | B / C.I (72 - 108) h | | | C.II / C.III (120 - 156) h | | |
| --- | --- | --- | --- | --- | --- | --- | --- | --- | --- |
|  | MV | SD | Comment | MV | SD | Comment | MV | SD | Comment |
| REF | 8.72 | 3.74 | insignificant | 3.82 | 0.06 | significant, * | 1.22 | 0.25 | insignificant |
| MTA | 6.64 | 0.06 |  | 9.73 | 2.26 |  | 3.07 | 3.52 |  |

Table S5. Metabolic model and carbon atom transition model.

| **ID** | **Enzyme name** | **Reaction and carbon atom transition** |
| --- | --- | --- |
| tGln | Glutamine importer | Gln_ex[(1,2,3,4,5)] => Gln[(1,2,3,4,5)] |
| gs | Glutaminase | Gln[(1,2,3,4,5)] => Glu[(1,2,3,4,5)] |
| tGlu | Glutamate transporter | Glu[(1,2,3,4,5)] = Glu_ex[(1,2,3,4,5)] |
| gdh | Glutamate dehydrogenase | aKG_m[(1,2,3,4,5)] = Glu_m[(1,2,3,4,5)] |
| tGlc | Glucose importer | Glc_ex[(1,2,3,4,5,6)] => G6P[(1,2,3,4,5,6)] |
| fGlyco | Carbon storage (glycogen) degradation | Glyco_ex[(1,2,3,4,5,6)] => G6P[(1,2,3,4,5,6)] |
| pgi | Phospho-glucose-isomerase | G6P[(1,2,3,4,5,6)] = F6P[(1,2,3,4,5,6)] |
| pfk | Phosphofructokinase + fructose-1,6-bisphosphatase | F6P[(1,2,3,4,5,6)] = FBP[(1,2,3,4,5,6)] |
| fbpa | Fructose-bisphosphate aldolase | FBP[(1,2,3,4,5,6)] = DHAP[(3,2,1)] + GAP[(4,5,6)] |
| tpi | Triophosphate isomerase | DHAP[(1,2,3)] = GAP[(1,2,3)] |
| gapdh | GAP dehydrogenase + biphosphoglycerate mutase | GAP[(1,2,3)] = 3PG[(1,2,3)] |
| eno | Phosphoglycerate hydratase | 3PG[(1,2,3)] = PEP[(1,2,3)] |
| pkm | Pyruvate kinase | PEP[(1,2,3)] => Pyr[(1,2,3)] |
| ldh | Lactate dehydrogenase | Pyr[(1,2,3)] = Lac[(1,2,3)] |
| tLac | Lactate transporter | Lac[(1,2,3)] => Lac_ex[(1,2,3)] |
| G6Pdh | Glucose-6-phosphate dehydrogenase | G6P[(1,2,3,4,5,6)] => Ru5P [(2,3,4,5,6)] + CO2[(1)] |
| rpi | Ribulose-5-phosphate isomerase | Ru5P[(1,2,3,4,5)] = R5P[(1,2,3,4,5)] |
| tkt1 | Transketolase | R5P[(1,2,3,4,5)(6,7,8,9,10)] = S7P[(6,7,1,2,3,4,5)] + GAP[(8,9,10)] |
| tald | Transaldolase | E4P[(1,2,3,4)] + R5P[(5,6,7,8,9)] = F6P[(5,6,1,2,3,4)] + GAP[(7,8,9)] |
| tkt2 | Transketolae | S7P[(1,2,3,4,5,6,7)] + GAP[(8,9,10)] = E4P[(4,5,6,7)] + F6P[(1,2,3,8,9,10)] |
| tCO2 | CO_2_ evolution | CO2[(1)] => CO2_ex[(1)] |
| MPC1 | Pyruvate/H^+^ symporter | Pyr[(1,2,3)] => Pyr_m[(1,2,3)] |
| CIC | Citrate/Malate antiporter | Cit_m[(1,2,3,4,5,6)] + Mal[(7,8,9,10)] => Cit[(1,2,3,4,5,6)] + Mal_m[(7,8,9,10)] |
| DIC | PO_3_^4-^/Malate antiporter | Mal_m[(1,2,3,4)] = Mal[(1,2,3,4)] |
| GC1 | Glutamate/H^+^ symporter | Glu_m[(1,2,3,4,5)] = Glu[(1,2,3,4,5)] |
| OGC | Malate/aKG antiporter | Mal[(1,2,3,4)] + aKG_m[(5,6,7,8,9)] = Mal_m[(1,2,3,4)] + aKG[(5,6,7,8,9)] |
| AGC1 | Aspartate/glutamate antiporter | Glu[(1,2,3,4,5)] + Asp_m[(6,7,8,9)] => Glu_m[(1,2,3,4,5)] + Asp[(6,7,8,9)] |
| mAla | Unknown alanine transporter | Ala[(1,2,3)] = Ala_m[(1,2,3)] |
| mAsn | Unknown asparagine transporter | Asn[(1,2,3,4)] = Asn_m[(1,2,3,4)] |
| pdh | Pyruvate dehydrogenase | Pyr_m[(1,2,3)] => AcCoA_m[(2,3)] + CO2[(1)] |
| cs | Citrate synthase | OAA_m[(1,2,3,4)] + AcCoA_m[(5,6)] => Cit_m[(4,3,2,6,5,1)] |
| idh | Iso-citrate dehydrogenase | Cit_m[(1,2,3,4,5,6)] = aKG_m[(1,2,3,4,5)] + CO2[(6)] |
| adh | aKG dehydrogenase + succinyl-CoA ligase + succinate dehydrogenase + | aKG_m[(1,2,3,4,5)(6,7,8,9,10)] = Fum_m[(2,3,4,5)(10,9,8,7)] + CO2[(1)(6)] |
| fus | fumarase | Fum_m[(1,2,3,4)(5,6,7,8)] = Mal_m[(1,2,3,4)(8,7,6,5)] |
| mdh | Malate dehyrogenase | Mal_m[(1,2,3,4)(5,6,7,8)] = OAA_m[(1,2,3,4)(8,7,6,5)] |
| pepck | Phosphoenolpyruvate carboxykinase | OAA[(1,2,3,4)] = PEP[(1,2,3)] + CO2[(4)] |
| me_c | Cytosolic malic enzyme | Mal[(1,2,3,4)] = Pyr[(1,2,3)] + CO2[(4)] |
| me_m | Mitochondrial malic enzyme | Mal_m[(1,2,3,4)] = Pyr_m[(1,2,3)] + CO2[(4)] |
| pc | Pyruvate carboxylase | Pyr_m[(1,2,3)] + CO2[(4)] = OAA_m[(1,2,3,4)] |
| mdh_c | Cytosolic malate dehydrogenase | OAA[(1,2,3,4)(5,6,7,8)] = Mal[(1,2,3,4)(8,7,6,5)] |
| tSer | Serine importer | Ser_ex[(1,2,3)] => Ser[(1,2,3)] |
| phdgh | Phosphoglycerate dehydrogenase + phosphoserine phosphatase | 3PG[(1,2,3)] => Ser[(1,2,3)] |
| sds | Serine dehydratase | Ser[(1,2,3)] => Pyr[(1,2,3)] |
| tAla | Alanine transporter | Ala[(1,2,3)] = Ala_ex[(1,2,3)] |
| alt_c | Cytosolic alanine aminotransferase | Pyr[(1,2,3)] + Glu[(4,5,6,7,8)] = Ala[(1,2,3)] + aKG[(4,5,6,7,8)] |
| alt_m | Mitochondrial alanine aminotransferase | Ala_m[(1,2,3)] + aKG_m[(4,5,6,7,8)] = Pyr_m[(1,2,3)] + Glu_m[(4,5,6,7,8)] |
| tAsp | Aspartate transporter | Asp_ex[(1,2,3,4)] = Asp[(1,2,3,4)] |
| tAsn | Asparagine importer | Asn_ex[(1,2,3,4)] => Asn[(1,2,3,4)] |
| ast_c | Cytosolic aspartate aminotransferase | Asp[(1,2,3,4)] + aKG[(5,6,7,8,9)] = OAA[(1,2,3,4)] + Glu[(5,6,7,8,9)] |
| ast_m | Mitochondrial aspartate aminotransferase | Asp_m[(1,2,3,4)] + aKG_m[(5,6,7,8,9)] = OAA_m[(1,2,3,4)] + Glu_m[(5,6,7,8,9)] |
| asns | Asparaginase | Asn_m[(1,2,3,4)] = Asp_m[(1,2,3,4)] |
| muG6P | Biomass formation from G6P | G6P[(1,2,3,4,5,6)] => G6P_X_ex[(1,2,3,4,5,6)] |
| muGAP | Biomass formation from GAP | GAP[(1,2,3)] => GAP_X_ex[(1,2,3)] |
| muRu5P | Biomass formation from Ru5P | R5P[(1,2,3,4,5)] => R5P_X_ex[(1,2,3,4,5)] |
| acl | Acetyl-CoA lyase | Cit[(1,2,3,4,5,6)] => OAA[(6,3,2,1)] + AcCoA[(5,4)] |
| muAcCoA | Biomass formation from AcCoA | AcCoA[(1,2)] => AcCoA_X_ex[(1,2)] |
| muSer | Biomass formation from serine | Ser[(1,2,3)] => Ser_X_ex[(1,2,3)] |
| muAla | Biomass formation from alanine | Ala[(1,2,3)] => Ala_X_ex[(1,2,3)] |
| muAsp | Biomass formation from aspartate | Asp[(1,2,3,4)] => Asp_X_ex[(1,2,3,4)] |
| muAsn | Biomass formation from asparagine | Asn[(1,2,3,4)] => Asn_X_ex[(1,2,3,4)] |
| muGln | Biomass formation from glutamine | Gln[(1,2,3,4,5)] => Gln_X_ex[(1,2,3,4,5)] |
| muGlu | Biomass formation from glutamate | Glu[(1,2,3,4,5)] => Glu_X_ex[(1,2,3,4,5)] |

Indices: c: cytosolic, m: mitochondrial, ex: extracellular


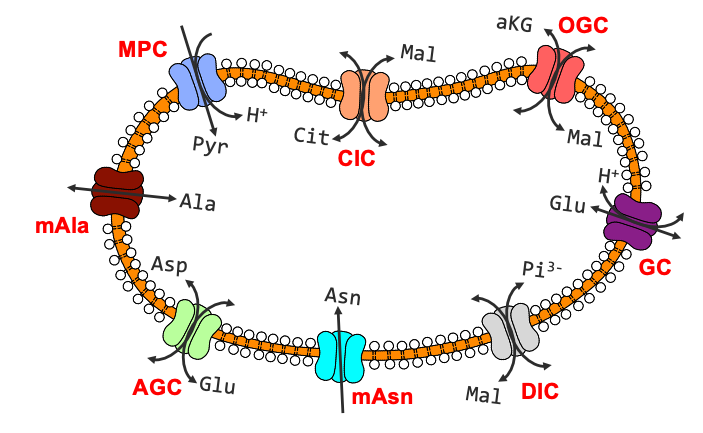


Figure S1. Mitchondrial transporters assumption in the metabolic model

Figure S2. IgG1 titer dynamics over the course of cultivation
