# Supplementary material for: Compartment‐specific 13C metabolic flux analysis reveals boosted NADPH availability coinciding with increased cell‐specific productivity for IgG1 producing CHO cells after MTA treatment
Source: Eng Life Sci. 2021 Nov 9;21(12):832–47. doi: 10.1002/elsc.202100057 (PMC8638276; doi:10.1002/elsc.202100057)

**** HEADER ****

Method: FBA

Mode: CPLEX

Model: Nielsen_CHO_FBA_simplified

Version: 4.3.0-SNAPSHOT

Date: 3/6/2020 - 8:05

Comment:

Objective: 1.0*m_NADPH 1.4037589999999995

Constraints:

-1.0*"Ex_D-Glucose" = 0.11284

1.0*Ex_Glycine <= 0.007123

1.0*Ex_Glycine >= 0.005828

1.0*Ex_Hypoxanthine = 0.0

1.0*"Ex_L-Alanine" <= 0.013079

1.0*"Ex_L-Alanine" >= 0.010701

-1.0*"Ex_L-Arginine" <= 0.00476

-1.0*"Ex_L-Arginine" >= 0.00389

-1.0*"Ex_L-Asparagine" <= 0.01907

-1.0*"Ex_L-Asparagine" >= 0.01561

-1.0*"Ex_L-Aspartate" <= 0.0047

-1.0*"Ex_L-Aspartate" >= 0.00384

-1.0*"Ex_L-Cysteine" <= 0.00452

-1.0*"Ex_L-Cysteine" >= 0.0037

1.0*"Ex_L-Glutamate" <= 0.008557

1.0*"Ex_L-Glutamate" >= 0.007001

-1.0*"Ex_L-Glutamine" <= 0.04954

-1.0*"Ex_L-Glutamine" >= 0.04054

-1.0*"Ex_L-Histidine" <= 0.00207

-1.0*"Ex_L-Histidine" >= 0.00169

-1.0*"Ex_L-Isoleucine" <= 0.00547

-1.0*"Ex_L-Isoleucine" >= 0.00448

1.0*"Ex_L-Lactate" <= 0.224561

1.0*"Ex_L-Lactate" >= 0.183732

-1.0*"Ex_L-Leucine" <= 0.00861

-1.0*"Ex_L-Leucine" >= 0.00704

-1.0*"Ex_L-Lysine" <= 0.00689

-1.0*"Ex_L-Lysine" >= 0.00564

-1.0*"Ex_L-Methionine" <= 0.00414

-1.0*"Ex_L-Methionine" >= 0.00339

-1.0*"Ex_L-Phenylalanine" <= 0.00347

-1.0*"Ex_L-Phenylalanine" >= 0.00284

-1.0*"Ex_L-Proline" <= 0.00349

-1.0*"Ex_L-Proline" >= 0.00286

-1.0*"Ex_L-Serine" <= 0.01893

-1.0*"Ex_L-Serine" >= 0.01548

-1.0*"Ex_L-Threonine" <= 0.00543

-1.0*"Ex_L-Threonine" >= 0.00445

-1.0*"Ex_L-Tryptophan" <= 0.00103

-1.0*"Ex_L-Tryptophan" >= 8.4E-4

1.0*"Ex_L-Tyrosine" = 0.0

-1.0*"Ex_L-Valine" <= 0.00519

-1.0*"Ex_L-Valine" >= 0.00425

1.0*tATP = 0.0

1.0*tCMP = 0.0

1.0*tCardiolipin = 0.0

1.0*tCholes = 0.0

1.0*tCholesterol = 0.0

1.0*tGMP = 0.0

1.0*tPhosphatidylglycerol = 0.0

1.0*tPhosphatidylinositol = 0.0

1.0*tPhosphatidylserine = 0.0

1.0*tSphingomyelin = 0.0

Equation: 0.11*D-Glucose + 0.91*H2O + 0.76E-2*O2 + 0.47E-2*L-Arginine + 0.19E-1*L-Asparagine + 0.47E-2*L-Aspartate + 0.45E-2*L-Cysteine + 0.49E-1*L-Glutamine + 0.2E-2*L-Histidine + 0.54E-2*L-Isoleucine + 0.86E-2*L-Leucine + 0.68E-2*L-Lysine + 0.41E-2*L-Methionine + 0.34E-2*L-Phenylalanine + 0.34E-2*L-Proline + 0.18E-1*L-Serine + 0.54E-2*L-Threonine + 0.1E-2*L-Tryptophan + 0.51E-2*L-Valine + 0.23E-1*Orthophosphate = 0.72*CO2 + 0.1E-2*Formate + 0.18*L-Lactate + 0.2*NH4 + 0.58E-2*Glycine + 0.1E-1*L-Alanine + 0.7E-2*L-Glutamate + 0.47E-2*Urea

**** EOF HEADER ****

**** Flux ****

2OXOADOXm 0.00103

2OXOADPTm 0.00103

3DSPHR 0.0

3HAO 0.00103

3SALATAi 0.00452

3SPYRSP 0.00452

AASS 0.00689

ACACT1r 0.0

ACACT1rm -0.00103

ACCOAC 0.0

ACITL 0.0

ACOATA 0.0

ACONTm 0.09142799999999995

ADK1 0.0

ADK3 0.0

ADKd 0.0

AKGDm 0.144287

ALATA_L -0.37300899999999987

ALATA_Lm 0.3633379999999999

AM6SAD 0.00103

AMCOXO 0.00103

AMPDA 0.23334699999999997

ARG 0.00476

ASNN 0.01907

ASPCTr 0.0

ASPGLUm 0.5336759999999998

ASPTA 0.32409899999999986

ASPTAm -0.5336759999999998

ATPtm 0.144287

Acyl-CoA_Syn 0.0

Alam -0.3633379999999999

BCAT1 0.00519

BCAT2 0.00861

BCAT3 0.00547

Biomass_pmol_cell 0.0

C14STRc 0.0

C3STDH1Pc 0.0

C3STKR2c 0.0

C4STMO1c 0.0

C4STMO2Pc 0.0

CBPS 0.0

CDIPTr_cho 0.0

CDS_cho 0.0

CEPTC_cho 0.0

CEPTE_cho 0.0

CHLP 0.0

CHLPCTD 0.0

CITtam 0.0

CO2m -0.3554109999999999

CSm 0.09142799999999995

CTPS2 0.0

CYSO 0.00452

CYTK1 0.0

CYTK10 0.0

CYTK11 0.0

CYTK12 0.0

DGK1 0.0

DHCR243r 0.0

DHCRD1_cho 0.0

DHCRD2_cho 0.0

DHFR 0.0

DHORTS 0.0

DMATTc 0.0

DPMVDc 0.0

DSAT_cho 0.0

DTMPK 0.0

EBP1c 0.0

ECOAH1m -0.00103

ENO 0.11284

ETC_1 0.0

ETC_2 0.0

ETHAK 0.0

Ex_CO2 0.7277199999999999

Ex_Choline 0.0

Ex_D-Glucose -0.11284

Ex_Ethanolamine 0.0

Ex_Formate 0.00103

Ex_Glycine 0.005828

Ex_H2O -0.9194179999999997

Ex_Hypoxanthine 0.0

Ex_L-Alanine 0.010701

Ex_L-Arginine -0.00476

Ex_L-Asparagine -0.01907

Ex_L-Aspartate -0.0047

Ex_L-Cysteine -0.00452

Ex_L-Glutamate 0.007001

Ex_L-Glutamine -0.04954

Ex_L-Histidine -0.00207

Ex_L-Isoleucine -0.00547

Ex_L-Lactate 0.183732

Ex_L-Leucine -0.00861

Ex_L-Lysine -0.00689

Ex_L-Methionine -0.00414

Ex_L-Phenylalanine -0.00347

Ex_L-Proline -0.00349

Ex_L-Serine -0.01893

Ex_L-Threonine -0.00543

Ex_L-Tryptophan -0.00103

Ex_L-Tyrosine 0.0

Ex_L-Valine -0.00519

Ex_NH4 0.20092999999999997

Ex_O2 -0.0076100000000000004

Ex_myo-Inositol 0.0

External_Pi -0.023780000000000023

FA16BS 0.0

FA180ACPH 0.0

FACOAL160i 0.0

FAS180ACP 0.0

FBA 0.0

FKYNH 0.00103

FOLR2 0.0

FTHFDH 0.006607999999999999

FUMc 0.24614699999999998

FUMm 0.144287

G3PD1 0.0

G5SADr -0.00349

G5SADrm 0.0

G6PDH2r 0.33852

GALU 0.0

GAPD 0.11284

GHMT2r 3.979999999999999E-4

GK1 0.0

GLNtm 0.04954

GLUDxm -0.11644899999999997

GLUN 0.04954

GLUTCOADHm 0.00103

GLUt2m -0.5293269999999999

GMPS2 0.0

GND 0.33852

GRTTc 0.0

Glyc_Syn 0.0

H2Om -0.169836

HACD1m -0.00103

HAL 0.00207

HEX1 0.11284

HKYNH 0.00103

HMGCOARc 0.0

HMGCOASi 0.0

HXACPHY 0.0

HXPRT 0.0

ICDHxm 0.09142799999999995

IMPD 0.0

INSK 0.0

IPDDI 0.0

IgG 0.0

KYN3OX 0.00103

LDH_L 0.183732

LNS14DMc 0.0

LNSTLSc 0.0

LSTO1c 0.0

LTAE 0.00543

M 0.0

MALtm -0.144287

MAT 0.00414

MDH -0.32409899999999986

MDHm 0.6251039999999998

ME1m -0.6251039999999998

ME2 0.7145329999999999

MEVK1c 0.0

MTHFC 0.006607999999999999

MTHFD 0.006607999999999999

NDPK1 0.23334699999999997

NDPK1m -0.144287

NDPK2 0.0

NDPK3 0.0

NDPK4 0.0

NDPK5 0.0

NDPK7 0.0

NDPK8 0.0

NH4m -0.06690899999999997

O2m 0.0

OMPDC 0.0

ORPT 0.0

P5CRxm 0.1780539999999999

PCLAD 0.00103

PDHm 0.031917999999999946

PETHCT 0.0

PFK 0.0

PGCD 0.0

PGI -0.22568

PGK 0.11284

PGL 0.33852

PGM 0.11284

PGMT 0.0

PGPPT_cho 0.0

PMEVKc 0.0

PPAP_cho 0.0

PPM 0.0

PPi_Hydro 0.0

PRO1xm 0.0

PROD2 0.00349

PROD2m 0.1780539999999999

PROtm 0.0

PRPPS 0.0

PSERT 0.0

PSP_L 0.0

PSSA1_cho 0.0

PUNP5 0.0

PYK 0.11284

PYRtm 0.29368399999999995

R00851 0.0

R01083 0.23334699999999997

R01135 0.23334699999999997

R01866 0.0

R02030 0.0

R02241 0.0

RE0453C 0.0

RE2410C 0.0

RE3347C -0.3361909999999999

RNDR2 0.0

RNDR3 0.0

RNDR4 0.0

RPE 0.22568

RPI 0.11284

SERPT 0.0

SMS_cho 0.0

SQLEc 0.0

SQLSc 0.0

SUCD1 0.00933

SUCD1m 0.144287

SUCOAS1m 0.144287

TALA 0.11284

TAT1 0.0

TAT2 0.00347

TKT1 0.11284

TKT2 0.11284

TMDS 0.0

TPI 0.0

TRDR 0.0

TRPO2 0.00103

UMPK 0.0

UMPK3 0.0

URIDK2 0.0

m_NADPH 1.4037589999999995

mu 0.0

qmab 0.0

r0060 0.014391999999999999

r0074 -0.00349

r0074m 0.0

r0512 0.0

r0911 0.0

r1290 0.3233709999999999

tATP 0.0

tCMP 0.0

tCardiolipin 0.0

tCholes 0.0

tCholesterol 0.0

tGMP 0.0

tPhosphatidylcholine 0.0

tPhosphatidylglycerol 0.0

tPhosphatidylinositol 0.0

tPhosphatidylserine 0.0

tSphingomyelin 0.0

tUMP 0.0

tUrea 0.00476

tdAMP 0.0

tdCMP 0.0

tdGMP 0.0

tdTMP 0.0

**** EOF Flux ****


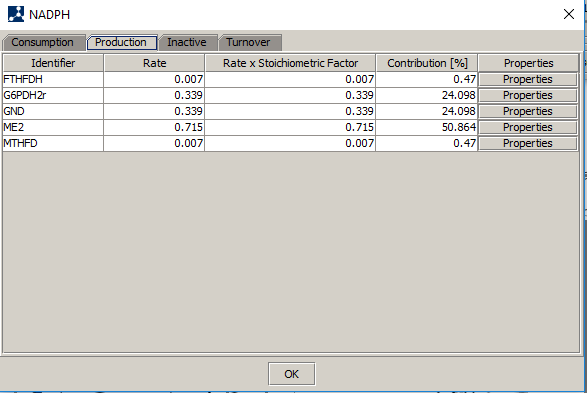

Supplement: Supplementary file 3 — Supporting information. [file ELSC-21-832-s004.docx]
