# Supplementary material for: Compartment‐specific 13C metabolic flux analysis reveals boosted NADPH availability coinciding with increased cell‐specific productivity for IgG1 producing CHO cells after MTA treatment
Source: Eng Life Sci. 2021 Nov 9;21(12):832–47. doi: 10.1002/elsc.202100057 (PMC8638276; doi:10.1002/elsc.202100057)
Supplement: Supplementary file 4 — Supporting information. [file ELSC-21-832-s002.pdf]

REFERENCE MID

MID	m+0			m+1			m+2			m+3			m+4			m+5			m+6		
	meas	meas_std	sim	meas	meas_std	sim	meas	meas_std	sim	meas	meas_std	sim	meas	meas_std	sim	meas	meas_std	sim	meas	meas_std	sim
G6P_T0	0.9300	0.0093	0.9300	0.0570	0.0006	0.0570	0.0120	0.0003	0.0120	0.0000	0.0010	0.0000	0.0000	0.0010	0.0000	0.0000	0.0010	0.0000	0.0000	0.0010	0.0000
G6P_T1	0.3290	0.0390	0.3431	0.1890	0.0270	0.2196	0.0760	0.0370	0.0321	0.0530	0.0150	0.0858	0.0400	0.0250	0.0284	0.0290	0.0250	0.0131	0.2830	0.0210	0.2770
G6P_T2	0.3100	0.0190	0.3422	0.1850	0.0250	0.2198	0.0670	0.0250	0.0323	0.0420	0.0250	0.0857	0.0330	0.0250	0.0287	0.0280	0.0250	0.0132	0.3350	0.0430	0.2771
P5P_T0	0.8430	0.0700	0.8430	0.1570	0.0016	0.1570	0.0000	0.0010	0.0000	0.0000	0.0010	0.0000	0.0000	0.0010	0.0000	0.0000	0.0010	0.0000			
P5P_T1	0.3360	0.0250	0.2751	0.0910	0.0250	0.1451	0.4270	0.0590	0.2192	0.0930	0.0250	0.1980	0.0000	0.0250	0.0602	0.0540	0.0760	0.1024			
P5P_T2	0.3620	0.0250	0.2727	0.0980	0.0250	0.1459	0.4000	0.1270	0.2192	0.0620	0.0250	0.1986	0.0000	0.0250	0.0609	0.0770	0.1090	0.1027			
DHAP_T0	1.0000	0.0100	1.0000	0.0000	0.0010	0.0000	0.0000	0.0010	0.0000	0.0000	0.0010	0.0000									
DHAP_T1	0.4990	0.0240	0.4956	0.1180	0.0250	0.1572	0.0720	0.0250	0.0711	0.3110	0.0250	0.2761									
DHAP_T2	0.4440	0.0270	0.4927	0.1230	0.0250	0.1589	0.1120	0.0250	0.0718	0.3210	0.0250	0.2765									
Pyr_T0	0.9090	0.0091	0.9090	0.0840	0.0008	0.0840	0.0070	0.0001	0.0070	0.0000	0.0010	0.0000									
Pyr_T1	0.7260	0.0250	0.7025	0.1180	0.0250	0.1085	0.0280	0.0250	0.0437	0.1290	0.0250	0.1453									
Pyr_T2	0.6270	0.0250	0.6558	0.1350	0.0250	0.1246	0.0390	0.0250	0.0517	0.1990	0.0250	0.1680									
Pyr_m_T0	0.9130	0.0091	0.9130	0.0800	0.0008	0.0800	0.0070	0.0001	0.0070	0.0000	0.0010	0.0000									
Pyr_m_T1	0.7440	0.0220	0.7293	0.1120	0.0250	0.1012	0.0260	0.0250	0.0391	0.1170	0.0250	0.1304									
Pyr_m_T2	0.6580	0.0330	0.6805	0.1260	0.0250	0.1174	0.0340	0.0250	0.0475	0.1810	0.0250	0.1546									
Ser_T0	0.9260	0.0093	0.9260	0.0650	0.0007	0.0650	0.0080	0.0001	0.0080	0.0000	0.0010	0.0000									
Ser_T1	0.9160	0.0230	0.9048	0.0590	0.0280	0.0466	0.0080	0.0250	0.0094	0.0170	0.0250	0.0382									
Ser_T2	0.8870	0.0180	0.9044	0.0550	0.0250	0.0469	0.0150	0.0250	0.0095	0.0420	0.0250	0.0383									
Ala_T0	0.9610	0.0096	0.9610	0.0370	0.0004	0.0370	0.0000	0.0010	0.0000	0.0020	0.0010	0.0020									
Ala_T1	0.8970	0.0340	0.8594	0.0590	0.0250	0.0659	0.0050	0.0250	0.0169	0.0400	0.0250	0.0578									
Ala_T2	0.8220	0.0290	0.8009	0.0640	0.0250	0.0828	0.0120	0.0250	0.0269	0.1020	0.0250	0.0893									
Ala_m_T0	0.9540	0.0095	0.9540	0.0410	0.0004	0.0410	0.0010	0.0000	0.0010	0.0040	0.0000	0.0040									
Ala_m_T1	0.9040	0.0100	0.8903	0.0530	0.0100	0.0576	0.0070	0.0250	0.0117	0.0360	0.0250	0.0405									
Ala_m_T2	0.8250	0.0330	0.8295	0.0690	0.0100	0.0746	0.0130	0.0250	0.0220	0.0930	0.0250	0.0739									
aKG_m_T0	0.9460	0.0095	0.9460	0.0540	0.0005	0.0540	0.0000	0.0010	0.0000	0.0000	0.0010	0.0000	0.0000	0.0010	0.0000	0.0000	0.0010	0.0000			
aKG_m_T1	0.7900	0.0600	0.7512	0.1090	0.0180	0.1316	0.0830	0.0410	0.1027	0.0120	0.0250	0.0142	0.0050	0.0250	0.0003	0.0050	0.0250	0.0000			
aKG_m_T2	0.6950	0.0680	0.7063	0.1350	0.0360	0.1551	0.1230	0.0480	0.1186	0.0300	0.0250	0.0197	0.0130	0.0250	0.0004	0.0130	0.0250	0.0000			
aKG_T0	0.9510	0.0095	0.9510	0.0490	0.0005	0.0490	0.0000	0.0010	0.0000	0.0000	0.0010	0.0000	0.0000	0.0010	0.0000	0.0000	0.0010	0.0000			
aKG_T1	0.8050	0.0250	0.7565	0.0970	0.0250	0.1294	0.0830	0.0250	0.1000	0.0090	0.0250	0.0138	0.0040	0.0250	0.0003	0.0010	0.0250	0.0000			
aKG_T2	0.6800	0.0220	0.7127	0.1290	0.0250	0.1523	0.1450	0.0250	0.1155	0.0310	0.0250	0.0191	0.0130	0.0250	0.0004	0.0020	0.0250	0.0000			
Fum_m_T0	0.9360	0.0094	0.9360	0.0640	0.0006	0.0640	0.0000	0.0010	0.0000	0.0000	0.0010	0.0000	0.0000	0.0010	0.0000						
Fum_m_T1	0.8950	0.0250	0.7890	0.0940	0.0250	0.1152	0.0000	0.0250	0.0938	0.0110	0.0250	0.0019	0.0000	0.0250	0.0000						
Fum_m_T2	0.7420	0.2010	0.7587	0.0930	0.0330	0.1297	0.1210	0.1710	0.1094	0.0270	0.0380	0.0022	0.0180	0.0250	0.0000						
Mal_m_T0	0.9670	0.0097	0.9670	0.0330	0.0003	0.0330	0.0000	0.0010	0.0000	0.0000	0.0010	0.0000	0.0000	0.0010	0.0000						
Mal_m_T1	0.8370	0.0250	0.7604	0.0770	0.0250	0.1200	0.0750	0.0250	0.0866	0.0110	0.0250	0.0315	0.0000	0.0250	0.0015						
Mal_m_T2	0.7580	0.0250	0.7264	0.1110	0.0250	0.1367	0.1050	0.0250	0.1009	0.0260	0.0250	0.0332	0.0000	0.0250	0.0029						
Mal_T0	0.9510	0.0095	0.9510	0.0360	0.0004	0.0360	0.0130	0.0001	0.0130	0.0000	0.0010	0.0000	0.0000	0.0010	0.0000						
Mal_T1	0.8250	0.0250	0.7451	0.0900	0.0250	0.1224	0.0730	0.0250	0.0833	0.0130	0.0250	0.0470	0.0000	0.0250	0.0022						
Mal_T2	0.7290	0.0226	0.7097	0.1240	0.0250	0.1400	0.1120	0.0250	0.0969	0.0310	0.0250	0.0492	0.0030	0.0250	0.0044						
Asn_T0	0.9360	0.0094	0.9360	0.0490	0.0005	0.0490	0.0120	0.0001	0.0120	0.0030	0.0000	0.0030	0.0000	0.0010	0.0000						
Asn_T1	0.9290	0.0110	0.9325	0.0370	0.0100	0.0504	0.0310	0.0300	0.0075	0.0030	0.0250	0.0092	0.0000	0.0250	0.0005						
Asn_T2	0.9290	0.0060	0.9262	0.0500	0.0100	0.0534	0.0150	0.0250	0.0090	0.0050	0.0250	0.0104	0.0000	0.0250	0.0011						
Asn_m_T0	0.9200	0.0092	0.9200	0.0600	0.0006	0.0600	0.0120	0.0001	0.0120	0.0080	0.0001	0.0080	0.0000	0.0010	0.0000						
Asn_m_T1	0.9070	0.0170	0.9154	0.0600	0.0250	0.0568	0.0230	0.0250	0.0121	0.0110	0.0250	0.0148	0.0000	0.0250	0.0009						
Asn_m_T2	0.8610	0.0320	0.9052	0.0670	0.0250	0.0616	0.0560	0.0280	0.0146	0.0170	0.0250	0.0168	0.0000	0.0250	0.0017						
Asp_T0	0.9460	0.0095	0.9460	0.0440	0.0004	0.0440	0.0100	0.0001	0.0100	0.0000	0.0010	0.0000	0.0000	0.0010	0.0000						
Asp_T1	0.8690	0.0215	0.8319	0.0810	0.0110	0.0817	0.0490	0.0250	0.0298	0.0000	0.0250	0.0539	0.0010	0.0250	0.0026						
Asp_T2	0.8160	0.0132	0.8142	0.1040	0.0100	0.0911	0.0770	0.0250	0.0342	0.0000	0.0250	0.0554	0.0030	0.0250	0.0051						
Asp_m_T0	0.9550	0.0096	0.9550	0.0410	0.0100	0.0410	0.0040	0.0100	0.0040	0.0000	0.0010	0.0000	0.0000	0.0010	0.0000						
Asp_m_T1	0.9270	0.0118	0.8965	0.0530	0.0100	0.0639	0.0200	0.0250	0.0173	0.0000	0.0250	0.0211	0.0000	0.0250	0.0012						
Asp_m_T2	0.8930	0.0100	0.8820	0.0700	0.0100	0.0708	0.0370	0.0250	0.0207	0.0000	0.0250	0.0240	0.0000	0.0250	0.0024						

MTA MID

MID	m+0			m+1			m+2			m+3			m+4			m+5			m+6		
	meas	meas_std	sim	meas	meas_std	sim	meas	meas_std	sim	meas	meas_std	sim	meas	meas_std	sim	meas	meas_std	sim	meas	meas_std	sim
G6P_T0	0.9540	0.0100	0.9540	0.0370	0.0100	0.0370	0.0090	0.0130	0.0090	0.0000	0.0100	0.0000	0.0000	0.0100	0.0000	0.0000	0.0100	0.0000	0.0000	0.0100	0.0000
G6P_T1	0.2230	0.0300	0.2556	0.1530	0.0250	0.2382	0.0910	0.0250	0.0581	0.0330	0.0100	0.0389	0.0320	0.0200	0.0485	0.0260	0.0100	0.0295	0.4420	0.0590	0.3314
G6P_T2	0.2360	0.0250	0.2556	0.1610	0.0250	0.2382	0.0910	0.0260	0.0581	0.0310	0.0100	0.0389	0.0300	0.0200	0.0485	0.0240	0.0100	0.0295	0.4260	0.0300	0.3314
P5P_T0	0.7580	0.0170	0.7580	0.2420	0.0170	0.2420	0.0000	0.0100	0.0000	0.0000	0.0100	0.0000	0.0000	0.0100	0.0000	0.0000	0.0100	0.0000			
P5P_T1	0.2400	0.0850	0.2098	0.0900	0.0300	0.1326	0.3630	0.1410	0.2185	0.1830	0.0200	0.1932	0.0000	0.0300	0.1031	0.1240	0.0390	0.1428			
P5P_T2	0.1720	0.0250	0.2097	0.1180	0.0250	0.1326	0.4660	0.0400	0.2185	0.1030	0.0750	0.1933	0.0000	0.0300	0.1031	0.1410	0.0400	0.1428			
DHAP_T0	0.9850	0.0210	0.9850	0.0150	0.0210	0.0150	0.0000	0.0100	0.0000	0.0000	0.0100	0.0000									
DHAP_T1	0.4760	0.0300	0.4138	0.1270	0.0250	0.1431	0.0570	0.0600	0.1298	0.3400	0.0250	0.3133									
DHAP_T2	0.4170	0.0100	0.4137	0.1200	0.0250	0.1431	0.1250	0.0100	0.1298	0.3380	0.0250	0.3134									
Pyr_T0	0.9090	0.0100	0.9090	0.0830	0.0100	0.0830	0.0070	0.0100	0.0070	0.0000	0.0100	0.0000									
Pyr_T1	0.7060	0.0190	0.7220	0.1220	0.0300	0.0792	0.0290	0.0250	0.0548	0.1430	0.0100	0.1430									
Pyr_T2	0.6170	0.0100	0.6184	0.1360	0.0250	0.1049	0.0410	0.0300	0.0771	0.2060	0.0100	0.1986									
Pyr_m_T0	0.9120	0.0100	0.9120	0.0780	0.0100	0.0780	0.0100	0.0100	0.0100	0.0000	0.0100	0.0000									
Pyr_m_T1	0.7360	0.0100	0.7375	0.1120	0.0250	0.0806	0.0290	0.0250	0.0532	0.1240	0.0100	0.1287									
Pyr_m_T2	0.6500	0.0150	0.6378	0.1240	0.0200	0.1071	0.0410	0.0250	0.0753	0.1840	0.0100	0.1799									
Ser_T0	0.9640	0.0100	0.9640	0.0310	0.0100	0.0310	0.0050	0.0100	0.0050	0.0000	0.0100	0.0000									
Ser_T1	0.9230	0.0250	0.8958	0.0440	0.0100	0.0435	0.0090	0.0100	0.0159	0.0240	0.0200	0.0448									
Ser_T2	0.8890	0.0100	0.8940	0.0550	0.0150	0.0440	0.0130	0.0100	0.0164	0.0432	0.0100	0.0456									
Ala_T0	0.9640	0.0100	0.9640	0.0340	0.0100	0.0340	0.0000	0.0100	0.0000	0.0020	0.0100	0.0020									
Ala_T1	0.8970	0.0200	0.8811	0.0470	0.0100	0.0503	0.0100	0.0250	0.0200	0.0480	0.0100	0.0486									
Ala_T2	0.7860	0.0100	0.7958	0.0710	0.0100	0.0697	0.0150	0.0250	0.0394	0.1270	0.0250	0.0951									
Ala_m_T0	0.9580	0.0100	0.9580	0.0350	0.0100	0.0350	0.0020	0.0100	0.0020	0.0040	0.0100	0.0040									
Ala_m_T1	0.8890	0.0100	0.8985	0.0580	0.0150	0.0461	0.0080	0.0100	0.0162	0.0450	0.0100	0.0383									
Ala_m_T2	0.7900	0.0250	0.8175	0.0760	0.0150	0.0643	0.0170	0.0200	0.0347	0.1170	0.0250	0.0825									
aKG_m_T0	0.9370	0.0100	0.9370	0.0610	0.0100	0.0610	0.0020	0.0100	0.0020	0.0000	0.0100	0.0000	0.0000	0.0100	0.0000	0.0000	0.0100	0.0000			
aKG_m_T1	0.7730	0.0100	0.7753	0.0970	0.0200	0.1158	0.1090	0.0200	0.0922	0.0140	0.0100	0.0164	0.0040	0.0100	0.0003	0.0030	0.0100	0.0000			
aKG_m_T2	0.6330	0.0300	0.6942	0.1370	0.0150	0.1516	0.1730	0.0300	0.1264	0.0390	0.0150	0.0273	0.0150	0.0150	0.0005	0.0020	0.0100	0.0000			
aKG_T0	0.9520	0.0100	0.9520	0.0480	0.0100	0.0480	0.0000	0.0100	0.0000	0.0000	0.0100	0.0000	0.0000	0.0100	0.0000	0.0000	0.0100	0.0000			
aKG_T1	0.7800	0.0250	0.8300	0.1070	0.0150	0.0950	0.1000	0.0250	0.0635	0.0120	0.0100	0.0112	0.0020	0.0100	0.0002	0.0000	0.0100	0.0000			
aKG_T2	0.6380	0.0370	0.7728	0.1380	0.0150	0.1203	0.1700	0.0250	0.0877	0.0370	0.0200	0.0188	0.0150	0.0150	0.0003	0.0020	0.0100	0.0000			
Fum_m_T0	0.9350	0.0100	0.9350	0.0650	0.0100	0.0650	0.0000	0.0100	0.0000	0.0000	0.0100	0.0000	0.0000	0.0100	0.0000						
Fum_m_T1	0.7790	0.0250	0.8065	0.1150	0.0100	0.1064	0.0000	0.0300	0.0854	0.0700	0.0400	0.0017	0.0360	0.0300	0.0000						
Fum_m_T2	0.7220	0.0200	0.7416	0.1480	0.0100	0.1364	0.0130	0.0300	0.1197	0.0920	0.0400	0.0024	0.0250	0.0200	0.0000						
Mal_m_T0	0.9630	0.0100	0.9630	0.0370	0.0100	0.0370	0.0000	0.0100	0.0000	0.0000	0.0100	0.0000	0.0000	0.0100	0.0000						
Mal_m_T1	0.7880	0.0150	0.7752	0.1110	0.0100	0.1086	0.0860	0.0100	0.0766	0.0150	0.0250	0.0377	0.0000	0.0100	0.0020						
Mal_m_T2	0.6720	0.0250	0.6934	0.1540	0.0100	0.1425	0.1360	0.0200	0.1077	0.0350	0.0150	0.0513	0.0030	0.0100	0.0051						
Mal_T0	0.9550	0.0100	0.9550	0.0370	0.0100	0.0370	0.0080	0.0100	0.0080	0.0000	0.0100	0.0000	0.0000	0.0100	0.0000						
Mal_T1	0.8030	0.0250	0.7711	0.1080	0.0100	0.1096	0.0750	0.0100	0.0759	0.0140	0.0250	0.0411	0.0000	0.0100	0.0023						
Mal_T2	0.6920	0.0100	0.6880	0.1490	0.0100	0.1435	0.1210	0.0200	0.1068	0.0330	0.0250	0.0559	0.0050	0.0100	0.0058						
Asn_T0	0.9380	0.0100	0.9380	0.0500	0.0100	0.0500	0.0120	0.0100	0.0120	0.0000	0.0100	0.0000	0.0000	0.0100	0.0000						
Asn_T1	0.9420	0.0250	0.9208	0.0490	0.0100	0.0529	0.0080	0.0100	0.0128	0.0010	0.0100	0.0130	0.0000	0.0100	0.0006						
Asn_T2	0.9330	0.0250	0.9024	0.0510	0.0100	0.0600	0.0090	0.0100	0.0181	0.0070	0.0100	0.0177	0.0000	0.0100	0.0017						
Asn_m_T0	0.9080	0.0280	0.9080	0.0600	0.0100	0.0600	0.0240	0.0130	0.0240	0.0080	0.0110	0.0080	0.0000	0.0100	0.0000						
Asn_m_T1	0.9030	0.0250	0.9157	0.0620	0.0100	0.0546	0.0270	0.0150	0.0144	0.0080	0.0100	0.0146	0.0000	0.0100	0.0007						
Asn_m_T2	0.8860	0.0250	0.8950	0.0640	0.0100	0.0626	0.0410	0.0200	0.0205	0.0080	0.0150	0.0200	0.0000	0.0100	0.0019						
Asp_T0	0.9480	0.0100	0.9480	0.0430	0.0100	0.0430	0.0090	0.0100	0.0090	0.0000	0.0100	0.0000	0.0000	0.0100	0.0000						
Asp_T1	0.8760	0.0100	0.8786	0.0770	0.0100	0.0676	0.0460	0.0250	0.0260	0.0000	0.0250	0.0265	0.0010	0.0100	0.0012						
Asp_T2	0.8050	0.0300	0.8407	0.1070	0.0250	0.0824	0.0840	0.0300	0.0371	0.0000	0.0300	0.0363	0.0040	0.0100	0.0035						
Asp_m_T0	0.9580	0.0100	0.9580	0.0400	0.0100	0.0400	0.0030	0.0100	0.0030	0.0000	0.0100	0.0000	0.0000	0.0100	0.0000						
Asp_m_T1	0.9190	0.0300	0.8827	0.0560	0.0100	0.0657	0.0250	0.0100	0.0255	0.0000	0.0250	0.0258	0.0000	0.0100	0.0012						
Asp_m_T2	0.8740	0.0250	0.8464	0.0770	0.0100	0.0799	0.0480	0.0100	0.0361	0.0000	0.0300	0.0352	0.0010	0.0100	0.0034						
